# Integrated drug response prediction models pinpoint repurposed drugs with effectiveness against rhabdomyosarcoma

**DOI:** 10.1371/journal.pone.0295629

**Published:** 2024-01-26

**Authors:** Bin Baek, Eunmi Jang, Sejin Park, Sung-Hye Park, Darren Reece Williams, Da-Woon Jung, Hyunju Lee

**Affiliations:** 1 School of Electrical Engineering and Computer Science, Gwangju Institute of Science and Technology, Gwangju, Republic of Korea; 2 School of Life Sciences, Gwangju Institute of Science and Technology, Gwangju, Republic of Korea; 3 Department of Pathology, Seoul National University Hospital, Seoul National University College of Medicine, Seoul, Republic of Korea; 4 Institute of Neuroscience, Seoul National University Hospital, Seoul, Republic of Korea; 5 Artificial Intelligence Graduate School, Gwangju Institute of Science and Technology, Gwangju, Republic of Korea; Children’s Cancer Institute Australia, AUSTRALIA

## Abstract

Targeted therapies for inhibiting the growth of cancer cells or inducing apoptosis are urgently needed for effective rhabdomyosarcoma (RMS) treatment. However, identifying cancer-targeting compounds with few side effects, among the many potential compounds, is expensive and time-consuming. A computational approach to reduce the number of potential candidate drugs can facilitate the discovery of attractive lead compounds. To address this and obtain reliable predictions of novel cell-line-specific drugs, we apply prediction models that have the potential to improve drug discovery approaches for RMS treatment. The results of two prediction models were ensemble and validated via *in vitro* experiments. The computational models were trained using data extracted from the Genomics of Drug Sensitivity in Cancer database and tested on two RMS cell lines to select potential RMS drug candidates. Among 235 candidate drugs, 22 were selected following the result of the computational approach, and three candidate drugs were identified (NSC207895, vorinostat, and belinostat) that showed selective effectiveness in RMS cell lines *in vitro* via the induction of apoptosis. Our *in vitro* experiments have demonstrated that our proposed methods can effectively identify and repurpose drugs for treating RMS.

## 1 Introduction

Targeted therapy aims to identify and attack specific cancer cells without affecting normal cells. However, experiments of all available compounds for targeted therapy with less toxicity is expensive and time-consuming. Computational methods to reduce the number of potential drugs can overcome these limitations.

Recently, pharmacogenomics, which is central to target-based drug discovery, relies on integrating [1, 2] or fusing [3] omics data (such as genomic and transcriptional profiles) to uncover the molecular characteristics of diseases and link these characteristics to drugs via computational algorithms. Cancers arise both from the disruption of functional modules in the complex cellular network of genes, transcriptomes, and protein isoforms, and from single gene abnormalities [4]. Thus, combining multiple types of omics data and integrating high-throughput information, rather than considering genes individually, can facilitate cancer research [5]. Numerous studies have been integrating multi-omics data for drug repositioning and drug-response profiling; multi-omics late integration (MOLI), based on deep neural networks [1]; supervised-feature extraction, for classifying drugs as ‘resistant’ or ‘sensitive’ via triplet loss [6]; a weighted graph regularized matrix factorization (WGRMF) algorithm [7]; a genomic-landscape-guided drug response prediction algorithm [8]; and a novel heterogeneous network-based method for drug-response prediction [9].

Several large-scale *in vitro* drug screening databases, including the Genomics of Drug Sensitivity in Cancer (GDSC) [10], the US National Cancer Institute (NCI)-60 [11], the Cancer Cell Line Encyclopedia [12], and the Cancer Therapeutics Response Portal [13], provide drug sensitivity for cancer cell lines. Among them, GDSC, a public database of information on cancer-cell drug sensitivity and drug-response molecular markers, comprises multiple omics datasets (including data on cancer-gene somatic mutations, gene amplification and deletion, and transcription) for over 1,000 cell lines derived from different tumor types. In spite of these, the availability of pharmacogenomics data is limited [14]. This is because there are many combinations of drug and cell line whose drug sensitivity has not yet been confirmed.

Rhabdomyosarcoma (RMS) is a soft-tissue sarcoma most often affecting children. Tumors form mainly in the genitourinary region (the bladder and uterus; ca. 31%), head and neck (ca. 25%), and limbs (ca. 13%), although it has the potential to occur anywhere in the body [15, 16]. In the United States, RMS accounts for 3% of all cancer cases in children aged 0–14 and 1% in adolescents aged 15–19 [17]. RMS prognosis and treatment depend on its location, tumor size, and metastasis state. Combination therapy comprising surgery, chemotherapy (vincristine, actinomycin D, cyclophosphamide, and ifosfamide [16, 18, 19]), and radiation therapy, is conventionally used to treat RMS. As immunotherapy and targeted therapy, which exploits molecules related to cancer cell growth, division, and survival, are generally safer than radiotherapy or chemotherapy with fewer side effects, they are under investigation as potential treatment options [20]. Despite these multimodal therapies, RMS outcomes remain dismal over 30 years. The overall survival (OS) at 10 years for metastatic RMS patients between 1980–1989, 1990–1999, and 2000–2010 was 29.7%, 29.1%, and 27.5%, respectively [21]. Therefore, it is urgent to find new treatment strategies for RMS.

In this study, we aimed to identify and repurpose drugs for treating RMS, integrating the predictions of two predictive models to obtain reliable results. Using the information on cancer cell lines and 235 target-drug effects from GDSC, a large pharmacogenomic database, we examined the shared omics characteristics behind the similar target-drug responses of these cell lines. Two computational models were trained using drug-response information from the GDSC, and they were used to predict drug responses for the human embryonal RMS (RD) and human alveolar RMS (SJCRH30) cell lines. Of the 235 drugs, 22 drugs with consistent results and high predictive performance in two predictive models were selected. Ten of the selected drugs were FDA-approved. We then validated these 22 candidate drugs via *in vitro* experiments and screened for non-specific toxicity in normal cells. Finally, three potential drugs (NSC207895, vorinostat, and belinostat) for treating RMS cell lines were identified. Notably, belinostat was sensitive in the SJCRH30 cell line, contrary to previously published studies [22]. As a result, our experiments indicate that an ensemble of two drug response prediction algorithms can effectively identify and repurpose drugs for RMS treatment.

## 2 Materials and methods

### 2.1 Dataset and pre-processing

#### 2.1.1 Public pharmacogenomic studies

For more than 250 drugs, GDSC provides drug-sensitivity information in the form of half-maximal inhibitory concentration (IC50, the area under the drug-response curve), the most commonly used indicator of enzyme-inhibitor interactions [10]. Iorio et al. [22] utilized a computational approach named “logic optimization for binary input to continuous output (LOBICO)” to binarize the IC50 values for 265 anti-cancer compounds in the GDSC database. They then classified cell lines as either ’resistant’ or ’sensitive’ to these compounds. Excluding drugs for which the original and rescreened results differed, we used the remaining 235 drugs as targets for model training. The binarized data are available at https://ars.els-cdn.com/content/image/1-s2.0-S0092867416307462-mmc6.xlsx.

To train our predictive model, we used GDSC gene expression, copy number variation, and somatic mutation data downloaded via the PharmacoGx 1.14.2 [23] R package. The downloaded gene expression data were generated using Affymetrix HG-U219, which were previously normalized using the robust multi-array average (RMA) method [24] with BrainArray [25] chip description file, as detailed in Safikhani et al. [26], and then log transformed. To systematically compare and analyze the training and testing gene expression data from different platforms, the transcript IDs must first be remapped to the HUGO Gene Nomenclature Committee (http://www.genenames.org/) [27] gene symbols, using the org.Hs.eg.db (ver. 3.8.2) R package [28]. When the multiple transcript IDs were mapped to a single gene, the average transcript expression value was used. Finally, to reduce false discovery, only the top 20% of IDs those with high variance (ca. 3,000 genes) were retained.

The PharmacoGx R package [23] provides gene-level maximum and minimum copy numbers from the GDSC as integers. While the minimum and maximum copy numbers usually coincide, they can differ if there is a break-point in the gene. The estimated copy numbers were then divided by the copy number of the copy-neutral state. We defined the copy-neutral state as the average cell-line ploidy, which can be downloaded from the Catalog of Somatic Mutations in Cancer. We then log transformed the minimum and maximum copy numbers, and retained the value with a larger absolute estimate. The pre-processing of GDSC copy numbers was carried out according to Hossein et al. [1]. Next, we re-mapped the gene IDs to the HUGO gene symbols and binarized copy numbers at the gene level, assigning zero to copy-neutral genes and one to amplified or deleted genes.

The mutation data can be downloaded from ftp://ftp.sanger.ac.uk/pub/project/cancerrxgene/releases/release-7.0/WES_variants.xlsx. Silent mutations were filtered out, and only those affecting protein structure were used.

#### 2.1.2 Sarcoma cell lines

We generated whole genome sequencing (WGS) and whole transcriptome sequencing (WTS) data from two human sarcoma cell lines (for RD and SJCRH30). For RD and SJRCH30 cell lines, we refer to the cell line from GDSC as ‘GDSC-RD’ and ‘GDSC-SJCRH30’, and ours simply as ‘RD’ and ‘SJCRH30’.

WTS data were produced using the Illumina TruSeq stranded mRNA library kit and NovaSeq 6000 platform, creating a 101 bp read length and 59,707,568 total reads. We used STAR 2-pass [29] to map the fastq files to the reference (GRCh37.75.gtf), to obtain BAM files. Next, we used RSEM [30] to calculate the read count and transcripts per million (TPM) values per gene, which were then log2 transformed. We then excluded non-informative genes (those with minimal variation; ca. 80% of the genes).

WGS data were generated using the TruSeq DNA PCR Free library kit and NovaSeq 6000 platform, which had a 150 bp read length and 1,002,299,192 total reads. The paired-end sequence was mapped to the human genome (GRCh37) using Illumina Isaac aligner. Then, copy number changes were obtained using InfoGenomeR [31], which provides an integrated breakpoint-based model (generating optimal breakpoint graphs) of genome-wide genomesegment connectivity, incorporating data about cancer-sample purity and ploidy, allele-specific copy number alteration, and haploid genotype. As input files, to improve InfoGenomeR’s somatic copy number alteration detection accuracy, we used bin files from BIC-seq2 [32] and structural-variant raw files from Manta [33] and Delly [34]. However, given that no control cell lines are available, all operations were performed in tumor-only mode, using the default parameters. To compare our data with GDSC copy number data, platform-specific IDs were transformed into the corresponding official gene symbols, as described. Finally, the gene-level copy number estimates were binarized, with zero assigned to copy-neutral genes and one to deleted or amplified genes.

Mutation data were obtained using GATK Mutect2 [35]. Mutect2 calls somatic short mutations, including single nucleotide variants and small insertions and deletions. Since our cell lines did not have matched normal samples, Mutect2 was run in tumor-only mode with the default parameters. To remove germline variants, we used the Genome Aggregation Database (gnomAD) germline population resource ftp://gsapubftp-anonymous@ftp.broadinstitute.org/bundle/Mutect2, and Panel of Normals (PoN; 1000 Genomes Project) [36] was used as control samples. The output of the Mutect2 function CollectF1R2Counts was passed to the GATK LearnReadOrientationModel function to obtain the prior probability of read orientation artifacts. After mutations were called on a cell line, the supporting reads for a set number of known variant sites were summarized using GATK GetPileupSummaries. Contamination was estimated using GATK CalculateContamination. Finally, likely false-positive calls were filtered using GATK FilterMutectCalls.

### 2.2 Approaches for drug response prediction

**Fig 1:**
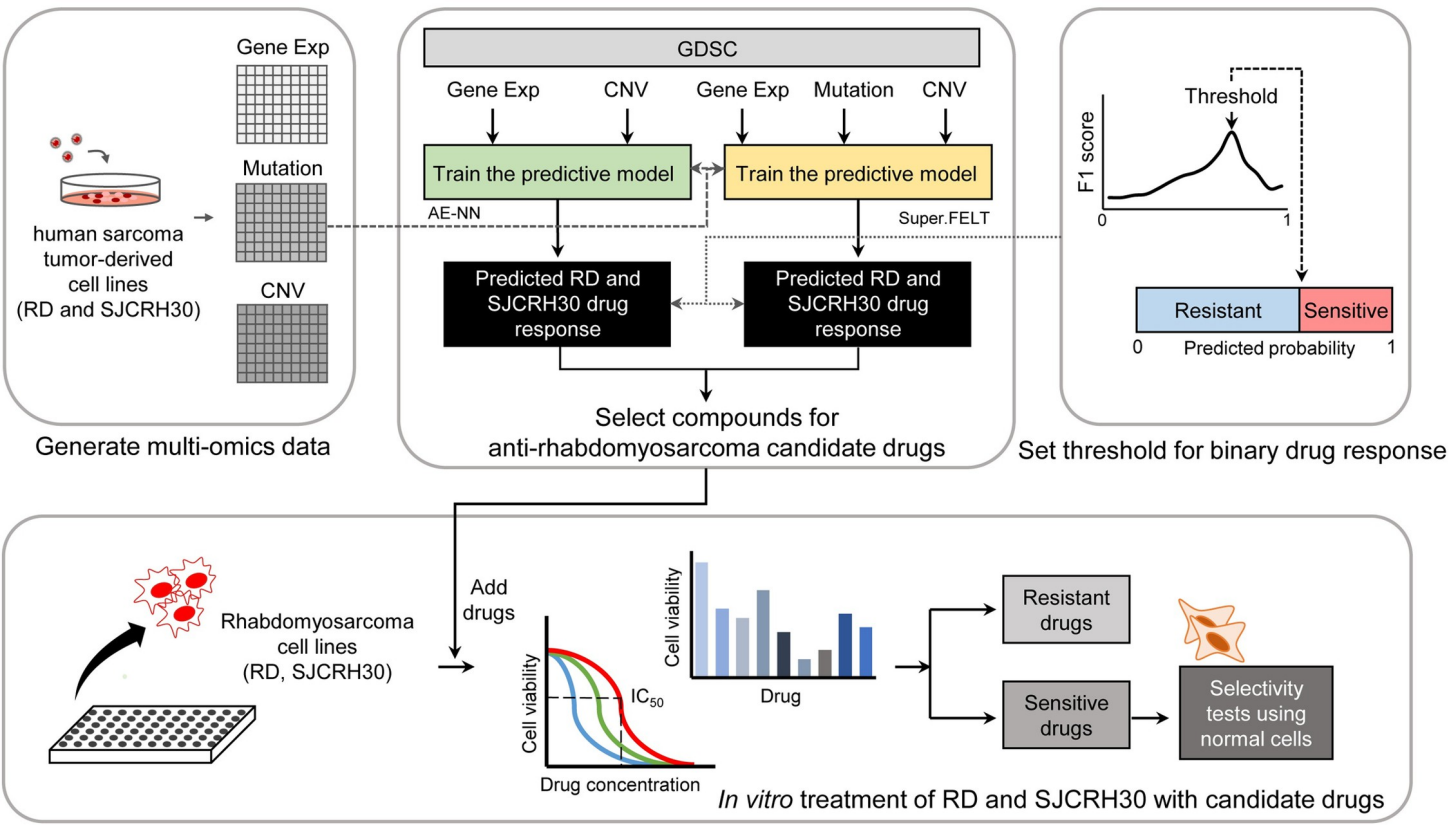
Overview of the analysis process. Gene Exp: gene expression; CNV: copy number variation; AE-NN: a predictive model consisting of an autoencoder and a fully connected neural network. Flowchart of drug response prediction process and drug screening. Genomics of Drug Sensitivity in Cancer (GDSC) multi-omics data were used to train the computational predictive models. The upper left corner illustrates multi-omics data of two generated human embryonal RMS (RD) and human alveolar RMS (SJCRH30) cell lines. The responses of the RD and SJCRH30 cell lines to all possible drugs were predicted using both trained models. The upper right shows that the predicted probability values are used to classify binary drug responses (’resistant’ and ’sensitive’). Finally, *in vitro* testing was conducted to validate RD and SJCRH30 responses to these potential candidate drugs (bottom of figure). Cell viability and IC_50_ were assessed via MTT assay. Drug resistance and sensitivity were verified. For testing the sensitivity eliciting drugs with high anti-cancer activity, we evaluated selectivity using the human colon fibroblast cell line (CCD-18Co) as a normal control, thereby validating the drug classifications.

We first trained a predictive model, comprising an autoencoder and fully-connected neural network classifier (AE-NN), using preprocessed GDSC gene expression and copy number data (upper middle of Fig 1). The Super.FELT [6] algorithm was trained using GDSC gene expression, copy number, and mutation data. The generated multi-omics data from the RMS cell lines RD and SJCRH30 (shown at the upper left of Fig 1) were tested in the two trained models (AE-NN and Super.FELT) to predict the response to each drug. After combining the results of these two models to identify candidate RMS drugs, the candidate drugs were *in vitro* validated in the RD and SJCRH30 cell lines (bottom of Fig 1).

#### 2.2.1 Drug response prediction models

The neural network-based prediction model consists of an autoencoder for dimensional reduction of the input omics data and a fully connected neural network with sigmoid function for binary drug response classification as ’resistant’ or ’sensitive’. The AE-NN model was trained using the GDSC gene expression and copy number data for 235 drugs. The GDSC dataset comprises approximately 20,000 genes. Since excessively high dimensionalities of datasets can impede model training, we applied variance filtering to reduce noise and model complexity. We then used an autoencoder to lower the dimensionality of the data while retaining as much information as possible. The AE-NN performed five-fold cross-validation (CV) [37] with 20% as test data and 80% as training data. Thresholds for binary drug response were set for each fold based on the highest F1 score [38] of the test data, where the F1 scores were calculated for every 0.01 thresholds between 0 and 1 (upper right of Fig 1). Then, the average threshold of the five folds was taken as the final threshold for each drug. The cell line response to each drug was categorized as ’sensitive’ if the predicted probability was greater than a threshold, and ’resistant’ if the predicted probability was less than a threshold. See S1 File and S1 Fig for model details.

Super.FELT [6] was trained using the GDSC omics gene expression, copy number, and mutation data for 230 drugs, using the hyperparameter tuning shown in S6 Table of the Super.FELT documentation. Super.FELT performed the five-fold CV (with 20% test and 80% training data), where test samples were used to decide the threshold for ‘resistant’ or ‘sensitive.’ Specifically, among thresholds obtained from the sklearn.metrics roc_curve function in the sklearn library, the threshold giving the highest F1 value was chosen for each fold. Then, five distinct thresholds were established from five folds, yielding five predicted responses for a given external test sample. The final response is subsequently determined based on the majority vote. Note that the GDSC-SJCRH30 cell line was included for AE-NN and Super.FELT training.

### 2.3 Cell culture

The RD (human embryonal rhabdomyosarcoma) and CCD-18Co (human colon fibroblast) cell lines were purchased from the Korean Cell Line Bank (KCLB). SJCRH30 (human alveolar rhabdomyosarcoma) cell line was purchased from the American Type Culture Collection (ATCC). RD and CCD-18Co cells were maintained in Dulbecco’s Modified Eagle’s Medium (DMEM; Gibco) supplemented with 10% fetal bovine serum (FBS; Gibco) and 1% penicillin/streptomycin (P/S; Gibco). SJCRH30 cells were maintained in Roswell Park Memorial Institute medium (RPMI 1640, Gibco) supplemented with 10% FBS and 1% P/S. Cells were grown in a humidified chamber containing 5% CO_2_ at 37°C. Cell line authentication was performed by short tandem repeat profile using PowerPlex® 18D system (Promega Corporation) and sequencing apparatus Applied Biosystems 3130xl Genetic Analyzer (Applied Biosystem) according to manufacturer’s protocol.

### 2.4 Cell viability assay

SJCRH30, RD, and CCD-18Co cells were seeded at 2 × 10^4^ cells/well in 96-well plates. The candidate drugs were treated for 24 h. AZ628, BIX02189, fedratinib, nutlin-3a, VX-11e, SB590885, NSC207895, tubastatin A, CX-5461, navitoclax, GSK1070916 and the FDA-approved drug library were purchased from Selleckchem. GW.44175 was purchased from Cayman chemical. MTT (3-(4,5-Dimethylthiazol-2-yl)-2,5-Diphenyltetrazolium bromide, Sigma) cell viability detection reagent diluted in serum free medium was added to each well and incubated for 2 h at 37°C. The supernatant was removed, and 50 μL DMSO was added to dissolve the precipitate. Absorbance was measured at 570 nm using a Molecular Devices VersaMax microplate reader and SoftMax® Pro 5 software. IC_50_ was calculated with GraphPad Prism 7 software.

### 2.5 Caspase-3/7 activity assay

The activity of caspase-3/7 was detected with the CellEvent^TM^ Caspase-3/7 Green detection kit (Thermo Fisher Scientific, C10423), following the manufacturer’s instructions. SJCRH30 and RD cells were seeded at 2 × 10^4^ cells/well in 96-well plates. Selected drugs (NSC207895, vorinostat, belinostat) were administered for 24 h and the cells were labeled with 10 μM of CellEvent^TM^ caspase-3/7 green detection reagent in serum free medium for 1 h at 37°C. Stained cells were observed under fluorescent microscopy (LEICA, DMI3000 B). Drugs were treated in triplicates and 4 pictures of each well were captured. The percentage of caspase-3/7 positive cells per field of view was quantified using ImageJ software.

### 2.6 Statistical analysis

Statistical significance for the cell culture experiments was determined using the Student’s *t*-test. A *P* value of less than 0.05 was considered as significant. The MTT assay and Caspase-3/7 activity assay were performed in triplicate. All data are expressed as the mean ± standard deviation.

## 3 Results

### 3.1 Prediction performance of the AE-NN model for GDSC cell lines

We constructed AE-NN prediction models for each of the 235 drugs using the GDSC cell line profiles, and measured the AUC and F1 scores to evaluate their prediction performance. The average five-fold cross-validation prediction performance of GDSC was 0.72 ± 0.09 for AUC and 0.42 ± 0.13 for F1 scores (S2 Table). For the top 10% of drugs ranked by AUC values, the average AUC value was as high as 0.86, which provides confidence for predicting drug responses for new cell lines using the constructed prediction models. In addition, for each of the 235 drug response prediction AE-NN models, a threshold for binarization of the prediction probabilities to sensitive and resistant was set as the value with the highest GDSC test F1 score (S2 Table).

### 3.2 Drug response prediction and drug selection for RMS cell lines

**Fig 2:**
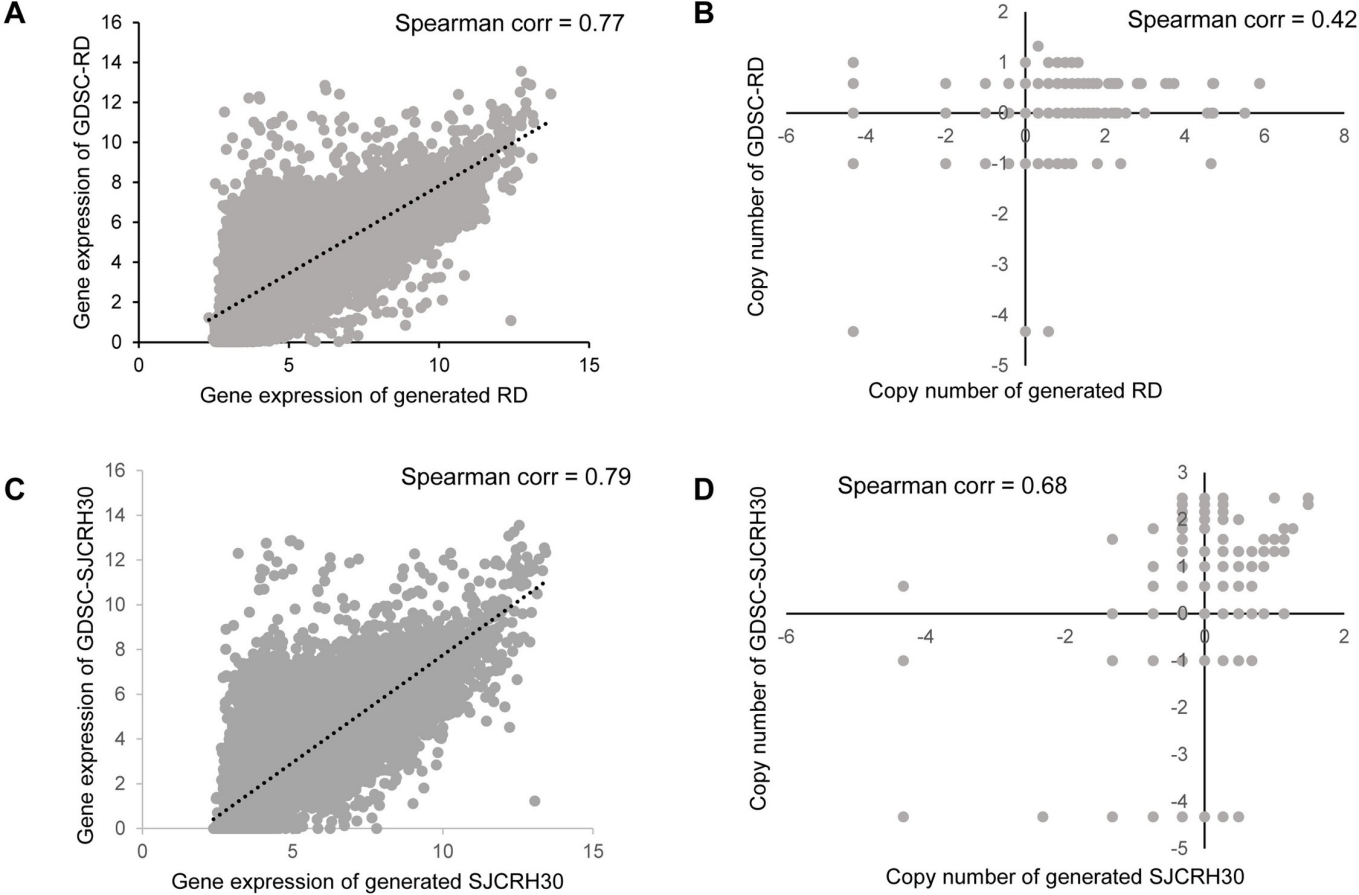
Comparison of RMS cell lines with that from the Genomics of Drug Sensitivity in Cancer (GDSC) database. corr: correlation. AE-NN: A predictive model comprising an autoencoder and a neural network classifier. (A) Spearman correlation of GDSC-RD and RD gene expression. (B) Spearman correlation of GDSC-RD and RD copy number. (C) Spearman correlation of GDSC-SJCRH30 and SJCRH30 gene expression. (D) Spearman correlation of GDSC- SJCRH30 and SJCRH30 copy number.

We predicted drug responses for the RD and SJCRH30 cell lines using the constructed AE-NN models. Among the 235 drugs, 12 and 223 drugs were predicted as sensitive and resistant, respectively (S2 Table). Then, we compared these predictions with the experiement results in GDSC data. Using GDSC data, Iorio et al. [22] classified the responses of 145 drugs for the RD cell line as sensitive or resistant, 11 eliciting sensitivity and 134 resistance; for SJCRH30 cell line, out of 196 drugs, 183 were resistant and 13 were sensitive (S2 Table). Spearman’s correlations between GDSC-RD and RD were 0.806 for gene expression and 0.416 for copy number (Fig 2A and 2B); Spearman’s correlations between GDSC-SJCRH30 and SJCRH30 were 0.793 for gene expression and 0.675 for copy number (Fig 2C and 2D). Although the gene expression data are correlated, the copy number showed relatively low correlation. Despite this, comparing the previously studied GDSC-RD drug-responses [22] and our AE-NN RD predictions for overlapping drugs, 140 of the 145 drugs showed consistent effects (S3 Table). Also previously studied GDSC-SJCRH30 drug-responses [22] and our AE-NN SJCRH30 predictions for overlapping drugs, 168 of the 196 drugs showed consistent effects (S3 Table).

We also predicted the RD and SJCRH30 cell lines using Super.FELT. Among the 230 drugs used for Super.FELT prediction, Super.FELT classified the responses of 116 drugs as sensitive and 114 as resistant for RD cell line; 121 drugs as sensitive and 109 drugs as resistant for SJCRH30 cell line (S2 Table). Note that Super.FELT classified more drugs as sensitive than AE-NN (S3 Table).

After predicting the SJCRH30 and RD cell lines, we sorted the drugs in decreasing order of AE- NN AUC value, selecting the five that elicited resistance, with the highest AUC values and consistent Super.FELT results, as ‘resistant’ candidates, and the five that elicited sensitivity, with high AUC values as ‘sensitive’ candidates, for *in vitro* testing. The ‘resistant’ candidates were methotrexate, AZ628, BIX02189, fedratinib (TG101348), and 5-fluorouracil, for the RD cell line (hereafter, ‘RD-R candidates’), and methotrexate, AZ628, BIX02189, fedratinib, and nutlin-3a, for the SJCRH30 cell line (‘SJ-R candidates’). The ‘sensitive’ candidates were trametinib, tubastatin-A, SB590885, QL-XI-92, and VX-11e, for the RD cell line (hereafter, ‘RD-S candidates’), and XMD14-99, KIN001-260, navitoclax (ABT-263), GSK1070916, and vorinostat, for the SJCRH30 cell line (‘SJ-S candidates’).

**Fig 3:**
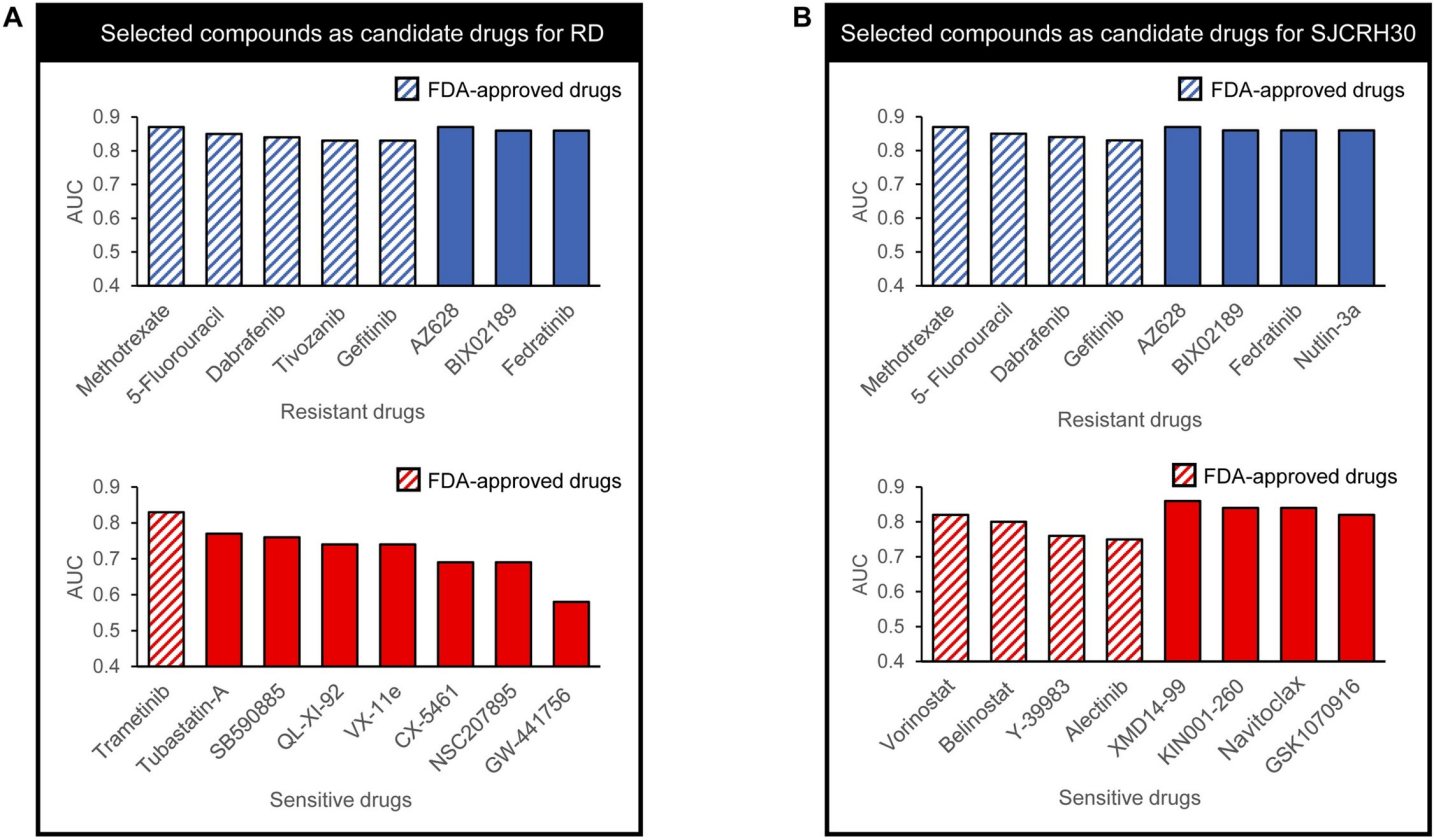
FDA-approved candidate drug selection. AUC; area under the curve of AE-NN. (A) Candidate drugs for the RD cell line. (B) Candidate drugs for the SJCRH30 cell line.

Some of these drugs, such as QL-XI-92 for RD-S and XMD14-99 and KIN001-260 for SJ-S, were not available for *in vitro* testing. Therefore, from those available for *in vitro* testing, we selected three more with FDA approval and/or high AUC values, for each group: dabrafenib, tivozanib (AV-951), and gefitinib, for RD-R; 5-fluorouracil, dabrafenib, and gefitinib, for SJ-R; CX-5461, NSC207895 (XI-006), and GW-441756, for RD-S; and belinostat (PXD101), Y-39983, and alectinib (CH5424802), for SJ-S. Those in the RD-R, SJ-R, and SJ-S groups are FDA-approved (Fig 3), while the RD-S drugs are not FDA-approved, but have high AUC values. Table 1 presents the AE-NN performances on the GDSC dataset for the candidate drugs.

**Table 1:** AE-NN performance of the selected candidate drugs.

**RD-R**	**Drug**	**AUC**	**F1**	**SJ-R**	**Drug**	**AUC**	**F1**
	Methotrexate	0.87	0.66		Methotrexate	0.87	0.66
	AZ628	0.87	0.60		AZ628	0.87	0.60
	BIX02189	0.86	0.64		BIX02189	0.86	0.64
	Fedratinib	0.86	0.63		Fedratinib	0.86	0.63
	5-Fluorouracil	0.85	0.58		Nutlin-3a	0.86	0.71
	Dabrafenib	0.84	0.58		5- Fluorouracil	0.85	0.58
	Tivozanib	0.83	0.54		Dabrafenib	0.84	0.58
	Gefitinib	0.83	0.61		Gefitinib	0.83	0.61
Avg	0.85 ±0.02	0.61 ±0.04	Avg		0.86 ±0.01	0.63 ±0.04
**RD-S**	Trametinib	0.83	0.68	**SJ-S**	XMD14-99	0.86	0.68
	Tubastatin-A	0.77	0.63		KIN001-260	0.84	0.69
	SB590885	0.76	0.50		Navitoclax	0.84	0.70
	QL-XI-92	0.74	0.63		GSK1070916	0.82	0.58
	VX-11e	0.74	0.41		Vorinostat	0.82	0.44
	CX-5461	0.49	0.74		Belinostat	0.80	0.44
	NSC207895	0.49	0.26		Y-39983	0.76	0.66
	GW-441756	0.58	0.31		Alectinib	0.75	0.50
	Avg	0.73 ±0.07	0.52 ±0.17		Avg	0.81 ±0.04	0.59±0.10

### 3.3 *In vitro* cell proliferation tests to validate the effect of the candidate drugs on RMS

**Fig 4:**
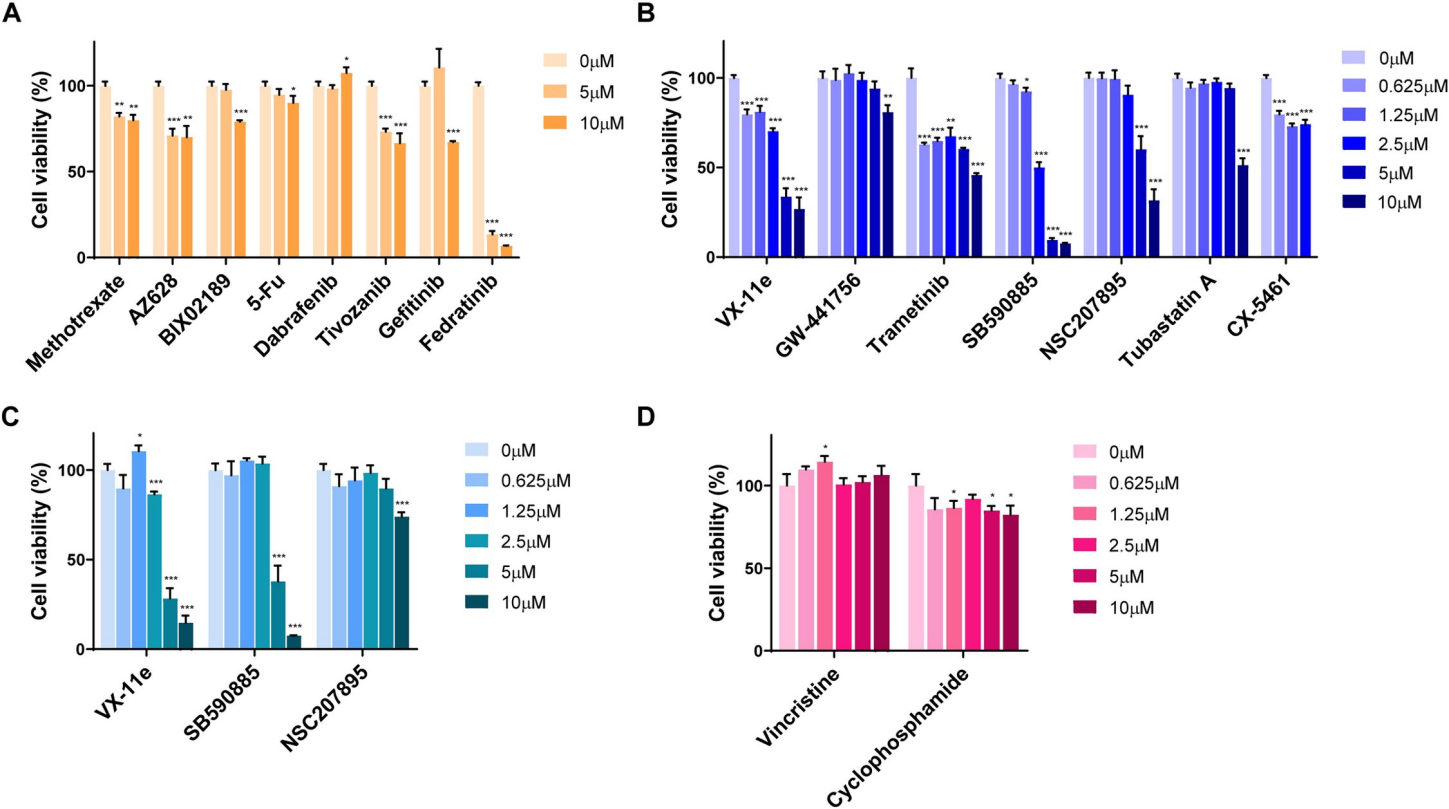
RD proliferation assay: Cell viability tests for the candidate drugs. (A) Human embryonal RMS cells were treated with RD-R drugs. (B) Human embryonal RMS cells were treated with RD-S drugs. (C) CCD-18Co normal human colon fibroblasts, as controls. (D) Comparison with the clinical drugs vincristine and cyclophosphamide. Data represent the mean ± SD. *P<0.05, **P<0.01, ***P<0.001 (*t-*test).

**Fig 5:**
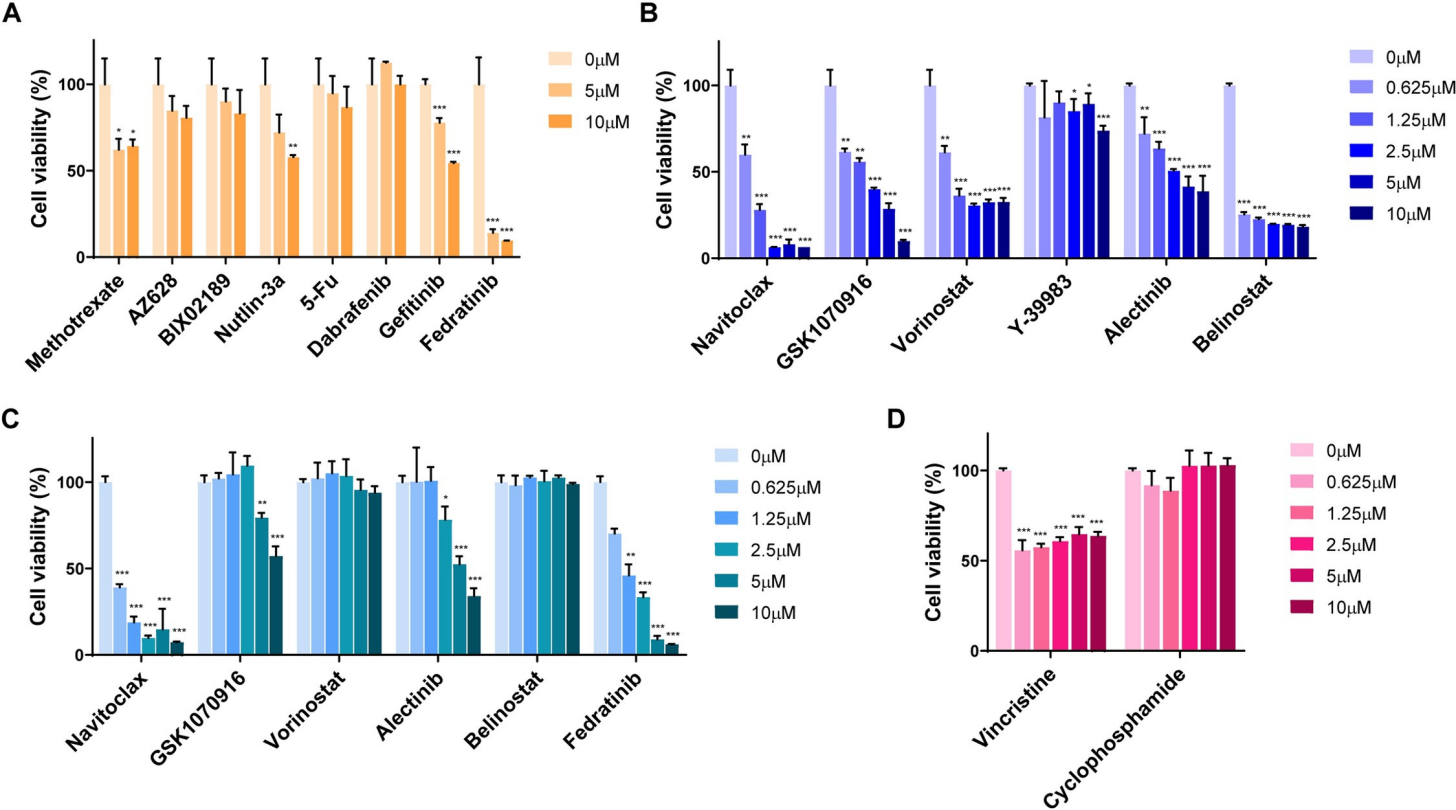
SJCRH30 proliferation assay: Cell viability tests for the candidate drugs. (A) Human alveolar RMS cells were treated with SJCRH30-R drugs. (B) Human alveolar RMS cells were treated with SJCRH30-S drugs. (C) CCD-18Co normal human colon fibroblasts, as controls. (D) Comparison with the clinical drugs vincristine and cyclophosphamide. Data represent the mean ± SD. *P<0.05, **P<0.01, ***P<0.001 (*t-*test).

To investigate the drug responses, we performed an MTT assay using the RMS cell lines (RD, SJCRH30), with a human colon fibroblast cell line (CCD-18Co) as the normal cell control. All of the resistance-eliciting drugs, except for fedratinib, showed low anti-proliferation activity in both RD and SJCRH30 cells (Figs 4A and 5A), while fedratinib showed high non-selective anti-proliferation activity, including toxicity toward the control line (Fig 5C). Of the sensitivity-eliciting drugs, VX-11e, SB590885, and NSC207895 showed higher anti-cancer activity in RD cells (Fig 4B). Navitoclax, GSK1070916, vorinostat, alectinib, and belinostat showed higher anti-cancer activity in SJCRH30 cells (Fig 5B). Based on the CCD-18Co control results, NSC207895, vorinostat, and belinostat have selective anti-cancer activity against the RMS cell lines (Figs 4C and 5C), with IC_50_ values of 6.566 μM, 0.8532 μM, and 0.1459 μM, respectively (Table 2).

**Table 2:** IC50 comparison with normal cells.

**RD-S**	**Drug**	**IC50(μM)**	
		**RMS**	**CCD-18Co**
	VX-11e	3.786	4.040
	SB590885	2.524	4.876
**SJ-S**	NSC207895	6.566	No cytotoxicity
	Navitoclax	0.764	0.319
	GSK1070916	1.397	No cytotoxicity
	Vorinostat	0.853	No cytotoxicity
	Alectinib	3.215	5.955
	Belinostat	0.146	No cytotoxicity

**Fig 6:**
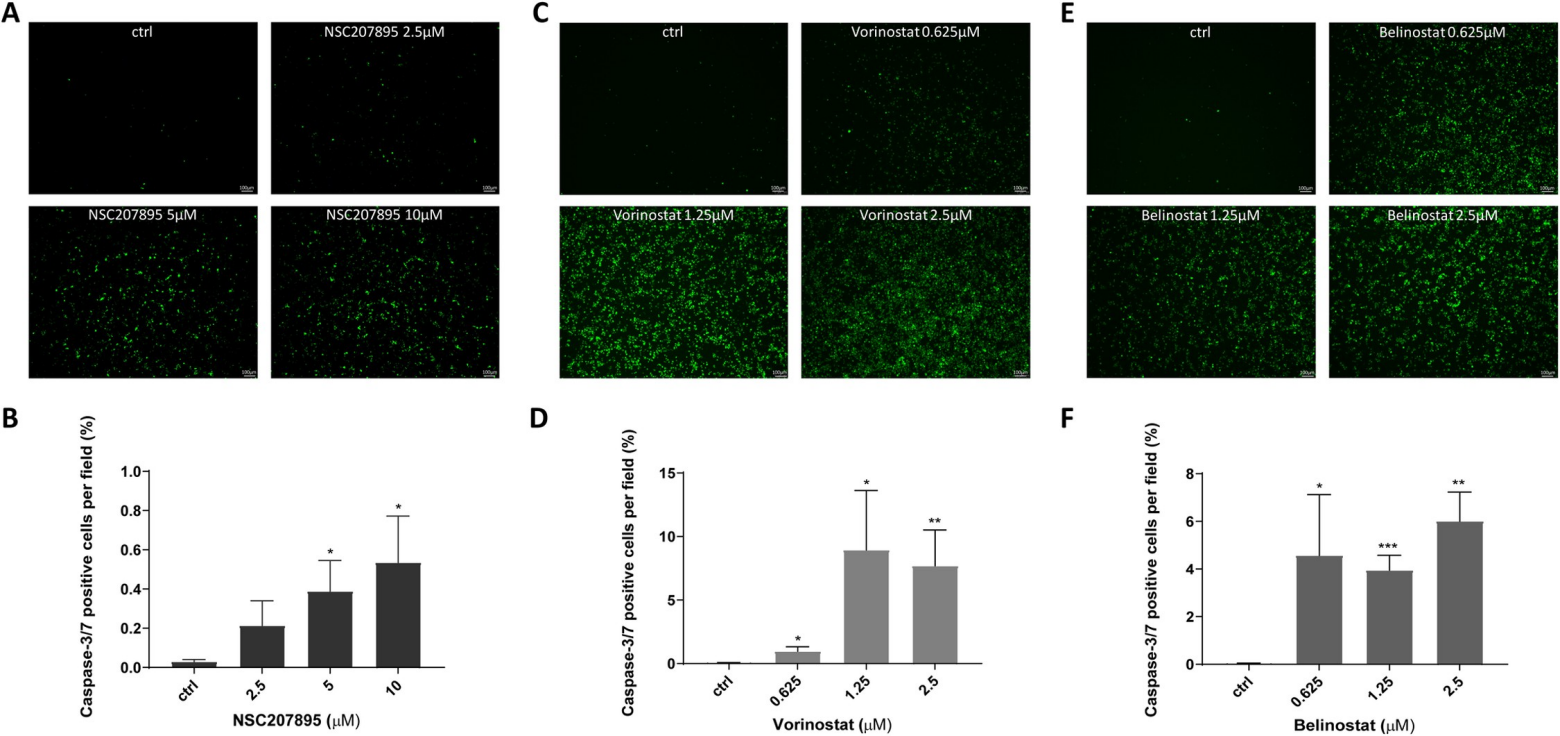
Caspase-3/7 activity assay for the validation of NSC207895, vorinostat and belinostat. (A) Representative images of caspase-3/7 activity after NSC207895 treatment in RD cells. (B) Quantification of the percentage of apoptotic cells per field of view. (C) Representative images of caspase-3/7 activity after vorinostat treatment in SJCRH30 cells. (D) Quantification of the percentage of apoptotic cells per field of view. (E) Representative images of caspase-3/7 activity after belinostat treatment in SJCRH30 cells. (F) Quantification of the percentage of apoptotic cells per field of view. Scale bar = 100 μm. Data represent the mean ± SD. *P<0.05, **P<0.01, ***P<0.001 (*t*-test).

Next, we compared the anti-cancer activity of the selected drugs and clinical drugs. The proliferation of each cell line after drug treatment was evaluated using the MTT assay. Vincristine and cyclophosphamide, which are clinically approved RMS treatments, were treated in each cell line for 24 h. In RD cells, NSC207895 effectively inhibited cell proliferation compared to vincristine and cyclophosphamide (Fig 4B and 4D). In SJCRH30 cells, vorinostat and belinostat also reduced cells proliferation to a greater degree than the clinical drugs (Fig 5B and 5D). Additionally, caspase-3/7 activities were evaluated for the validation of anti-cancer activity via apoptotic signaling. NSC207895 treatment significantly induced the activation of caspase-3/7 in RD cells does dependently (Fig 6A and 6B). Vorinostat and belinostat treatment also increased level of caspase-3/7 activation in SJCRH30 cells (Fig 6C–6F). Taken together, it was observed that NSC207895, vorinostat, and belinostat were selectively effective in rhabdomyosarcoma cells via the induction of apoptosis.

## 4 Discussion and conclusion

Drug-response prediction is vital for developing cancer therapeutics, such as the selection of effective drugs for sarcoma. Here, we apply a machine-learning model that combines an autoencoder and a neural network classifier to predict these drug responses. Our model generated a candidate drug list, with associated sarcoma resistance or sensitivity responses. We validated the anticancer effects via *in vitro* testing. The models were trained using the omics profiles of over 1,000 cell lines of various tumor types, and their responses to 235 drugs from the GDSC database. We then predicted candidate drug responses for the sarcoma tumor-derived cell lines, SJCRH30 and RD. To train the AE-NN predictive model, cell-line gene expression and copy number data were inputted into an autoencoder, and the abstract characteristics obtained from the auto-encoder were used as the input for a neural network classifier. The neural network classifier was used to predict binary cell-line responses to the drugs. For the Super.FELT algorithm, gene expression, copy number, and mutation data were used for training, which was then predicted. By integrating the results of each predictive model, we selected 22 candidate drugs for *in vitro* testing to validate their efficacy. In most cases, the experimental results were consistent with the predictions, although fedratinib had the opposite effect: predicted to elicit resistance, it elicited high *in vitro* sensitivity for both RD and SJCRH30. In contrast, the GDSC-RD cell line, which was not used for training, exhibited resistance to fedratinib [22]. *In vitro*, the CCD-18Co human colon fibroblast control line was highly sensitive to fedratinib. We therefore consider fedratinib highly toxic and difficult to use clinically. Additionally, the response of GDSC-SJCRH30 to belinostat reported by Iorio et al. [22] was ’resistant’, but predicted as ’sensitive’ by both AE-NN and Super.FELT, and have consistent ’sensitive’ results *in vitro* validation. Thus, we recommended belinostat as a non-toxic drug that could be used to treat sarcoma.

Since the SJCRH30 cell line was included in the AE-NN training process, we compared the results when the test cell line SJCRH30 was excluded from the model training process (result without GDSC-SJCRH30 are in S4 Table). Overall, the binary drug response prediction results did not change significantly because only one cell line SJCRH30 was missing from approximately 795 cell lines in each drug-specific training model. Three drugs that were predicted as RD-R but changed to RD-S and one drug that changed from RD-S to RD-R; one drug that changed from SJ-R to SJ-S and two drugs that changed from SJ-S to SJ-R. In these cases, i) the predicted probability is very close to the threshold so that the binary response changes due to very small differences, and/or ii) the results of drugs with low AUC of the model changed, indicating that the models for these drugs are not robust. In addition, the AE-NN classifier had three epochs per fold, and the average result of all epochs was used as the final result for each fold. Considering that the general prediction model uses only the results of the best epoch, not the average results of all epochs, we compare the results of the best epoch and average epoch (best epoch results are in S5 Table). There were a total of five drugs whose results changed. The results of the best epoch were quite similar to the results of the average epoch, but this is because the structure of the model was relatively simple and the number of epochs was small. Note that although the prediction results may vary depending on the training data and training epoch, ‘RD-R candidates’, ‘SJ-R candidates’, ‘RD-S candidates’ and ‘SJ-S candidates’ were remained the same in these different settings.

Integrating the AE-NN and Super.FELT predictions identified eight drugs that elicited sensitivity in each of RD and SJCRH30. Among the FDA-approved drugs, only trametinib elicited sensitivity in the RD cell line, whereas vorinostat, belinostat, Y-39983, and alectinib elicited sensitivity for SJCRH30. *In vitro* testing of the sensitivity eliciting drugs, NSC207895, vorinostat, and belinostat revealed RMS cell-line sensitivity. NSC-207895 is a MDMX inhibitor known to have antitumor activity [39]. Vorinostat and belinostat are both FDA-approved histone deacetylase (HDACs) inhibitor for cutaneous T-cell lymphoma (CTCL) and peripheral T-cell lymphoma (PTCL) treatment, respectively [40, 41]. To the best of our knowledge, few prior studies have validated that these three drugs target sarcoma. Specifically, for Ewing’s sarcoma, Pishas et al. [42] reported that NSC207895 induces p53-independent apoptosis. For a cohort of heavily pre-treated soft-tissue sarcoma patients, Schmitt et al. [43] reported a low objective response to vorinostat: 6 of the 40 patients showed long-term disease stabilization. Hrzenjak et al. [44] reported that vorinostat inhibited the uterine sarcoma growth *in vitro* and in vivo. Lastly, for soft tissue sarcoma, Vitfell-Rasmussen et al. reported that belinostat in combination with doxorubicin was superior to single-agent doxorubicin [45].

Consistency between datasets from different platforms and projects is of great importance. Here, the GDSC-derived training data and the two cell lines that we generated were from different platforms. Integrating pharmacogenomics data from different sources is challenging, and the compatibility of data from different microarray platforms has long been questioned [46]. Such problems arise because there is a lack of standardized protocols and annotation methods, including for handling noise. Similarly, drug responses in the Cancer Cell Line Encyclopedia, one of the most commonly used pharmacogenomics databases, are inconsistent with those in GDSC [26] and the Cancer Genome Project [47]. Geeleher et al. [48], however, has refuted the claim that the findings of Haibe-Kains et al. [47] are unsubstantiated, and have verified the correlation between these pharmacogenomics datasets. To address this problem, Smirnov et al. [23] also provided a unified framework for meta-analysis of data from large pharmacogenomic datasets. Nonetheless, further research into pharmacogenomic database consistency is required, which will give an opportunity to increase the performance of drug prediction models.

Although this study used simple neural network-based prediction models for drug response classification, other advanced prediction models can be applied by following our drug recommendation protocol, expecting that new candidate drugs for RMS. In summary, we indicated that NSC207895, vorinostat, and belinostat have selective anti-RMS effects against normal cell control. It is suggesting the possibility of a treatment that can alleviate the limitations of poor prognosis in aggressive RMS. Our trained model, which uses information about new cell lines to predict their responses to target drugs, has the potential to improve RMS treatment and outcomes.

## Supporting information

S1 File Neural network-based predictive model. (PDF)

S1 Fig Model architecture for predicting cell line response to drugs. The prediction model consists of an Autoencoder and a neural network (AE-NN). The Autoencoder reduces high-dimensional omics data to low-dimensional data. Gene expression and copy number data are each passed through the Autoencoder, and when the loss between the reconstructed data and the input is small enough, the hidden embedding layer (bottleneck) values of the two omics data are concatenated. The concatenated embedded matrix are passed through a neural network classifier, where the prediction probability is calculated and can be classified as ’Resistant’ or ’Sensitive’ based on a threshold. The predictive model is trained and tested with the GDSC gene expression and copy number data with five-fold cross-validation, and used to predict drug response in the sarcoma cell lines RD and SJCRH30.
(PDF)

S1 Table List of experimented drugs and the number of GDSC samples. (PDF)

S2 Table Predicted drug response of RD and SJCRH30 cell lines. Pred_RD and Pred_SJCRH30 are the predicted values of the RD and SJCRH30 cell lines mapped to probabilities using the sigmoid function of AE-NN. The predicted probabilities of each cell line were binarized based on the threshold in the last column of the table and inserted into the Response_RD and Response_SJCRH30 columns, respectively. The averaged predictive performance of the GDSC 5-fold cross-validation of AE-NN for each drug was evaluated with AUC and F1 scores, and each threshold was set as the value at the highest F1 score. Iorio_RD column is the published drug responses to RD cell line by Iorio et al. (2016). Of the 145 non-NA drugs with binarized drug responses in the Iorio_RD column, the responses of 140 drugs were consistent with the binarized prediction results of the AE-NN model (Response_RD column). The accuracy was 0.966, the sensitivity was 0.636, and the specificity was 0.993. Super.FELT_RD and Super.FELT_SJCRH30 are binarized prediction results by Super.FELT model of RD and SJCRH30 cell lines. The result of Super.FELT classifications of RD cell line were consistent with the Iorio’s for 57 of the 141 overlapped drugs. The accuracy was 0.404, the sensitivity was 0.8, and the specificity was 0.374.
(PDF)

S3 Table Confusion matrix. (PDF)

S4 Table Predicted drug response of RD and SJCRH30 cell lines (w/o RD and SJCRH30 from training data). (PDF)

S5 Table Predicted drug response of RD and SJCRH30 cell lines (best epoch result). (PDF)
