# Supplementary material for: Integrated drug response prediction models pinpoint repurposed drugs with effectiveness against rhabdomyosarcoma
Source: PLoS One. 2024 Jan 26;19(1):e0295629. doi: 10.1371/journal.pone.0295629 (PMC10817174; doi:10.1371/journal.pone.0295629)
Supplement: S1 File — (PDF) [file pone.0295629.s001.pdf]

## S1 Note. Neural network-based Predictive model.

The predictive model comprising an autoencoder and fully-connected neural network classifier (AE-NN) consists of an autoencoder for dimension reduction of input omics data, and a fully-connected neural network for binary drug response classification as 'resistant' or 'sensitive'. Detail of AE-NN is depicted in Supplementary Figure 1. Before training the model, an experimental design that considers biological characteristics is required. It is difficult to train a model using biological data because the number of available samples is much smaller than the number of features. The number of samples (cell lines) available to train the drug response prediction model varied from drug to drug, averaging 775.6. On average, 683.5 samples were 'resistant' and 91.2 were 'sensitive', indicating imbalanced ratios ranging from 1:0.01 to 1:1.36, with an average ratio of 1:0.14 (Supplementary Table S1). Binary classification imbalances tend to bias most classes. Classifiers typically address this by ignoring small classes, focusing on classifying large classes correctly [1]. To overcome this, when we used five-fold cross-validation (CV), where the GDSC samples were randomly divided into five equal subsamples (folds), we stratified the data into folds with data augmentation via random oversampling [2] of minority drug responses. Using five-fold CV, we used 20% as the test set and the remaining 80% as the training set. For each fold, the epoch of the classifier was three, and the average results of each epoch was used as the final result for each fold.

The GDSC dataset consists of approximately 20,000 genes. The dimensionality of the data was reduced before entering the classifier to reduce noise and complexity in model training. First, we filtered out about 10,000 genes by excluding genes with a variance less than 0.1 from the gene expression data and genes with a variance less than 0.2 from the copy number data. It should be noted that this number may vary depending on the drug being studied. Afterward, only the genes shared by both datasets are inputted into the autoencoder algorithm. The autoencoder is an unsupervised artificial neural network that compresses data into a lower dimension and reconstructs the input without losing information. It comprises an encoder-decoder and a bottleneck (hidden embedding layer), and includes batch normalization, dropout, and the Adam optimizer. Each encoder-decoder has hidden layers of 1024 units (or neurons) and 512 units. By calculating the Pearson correlation coefficient [3] between the input

matrix of the encoder and reconstructed matrix of the decoder, we can confirm how well the hidden embedding layer represents the characteristics of the input matrix. The use of Euclidean distances improves the reproduction of the reconstructed matrix. To accurately assess the predictive ability of the model, we excluded the GDSC-RD cell line from the training process.

Next, we concatenated the gene expression and copy number embedded matrices from the hidden embedding layer, generating a concatenated matrix, which is the main input for the classifier. Because each embedded matrix has a dimension of 512, the concatenated result has a dimension of 1024. The classifier is a two-layered neural network model with a ReLU activation function for the hidden layer, and a sigmoid activation function for the last layer of the classifier. The hidden layer has 512 neurons and the output layer has one neuron. We used binary cross-entropy for the loss function, and an Adam optimizer to adjust the parameters. The hyperparameters for learning rate and batch size were set as 0.01 and 54, respectively. The threshold used to classify the predicted probability (the last-layer outcomes) as 'resistant' or 'sensitive' was the value of having the highest GDSC test f1 score.

# Supplementary References


[1] S. Kotsiantis, D. Kanellopoulos, and P. Pintelas, "Handling imbalanced datasets: A review," *GESTS international transactions on computer science and engineering,* vol. 30, no. 1, pp. 25-36, 2006.

[2] G. Lemaître, F. Nogueira, and C. K. Aridas, "Imbalanced-learn: A python toolbox to tackle the curse of imbalanced datasets in machine learning," *The Journal of Machine Learning Research,* vol. 18, no. 1, pp. 559-563, 2017.

[3] J. Benesty, J. Chen, Y. Huang, and I. Cohen, "Pearson correlation coefficient," in *Noise reduction in speech processing*: Springer, 2009, pp. 1-4.
