# Supplementary material for: Integrated drug response prediction models pinpoint repurposed drugs with effectiveness against rhabdomyosarcoma
Source: PLoS One. 2024 Jan 26;19(1):e0295629. doi: 10.1371/journal.pone.0295629 (PMC10817174; doi:10.1371/journal.pone.0295629)
Supplement: S1 Fig — The prediction model consists of an Autoencoder and a neural network (AE-NN). The Autoencoder reduces high-dimensional omics data to low-dimensional data. Gene expression and copy number data are each passed through the Autoencoder, and when the loss between the reconstructed data and the input is small enough, the hidden embedding layer (bottleneck) values of the two omics data are concatenated. The concatenated embedded matrix are passed through a neural network classifier, where the prediction probability is calculated and can be classified as ’Resistant’ or ’Sensitive’ based on a threshold. The predictive model is trained and tested with the GDSC gene expression and copy number data with five-fold cross-validation, and used to predict drug response in the sarcoma cell lines RD and SJCRH30. (PDF) [file pone.0295629.s002.pdf]

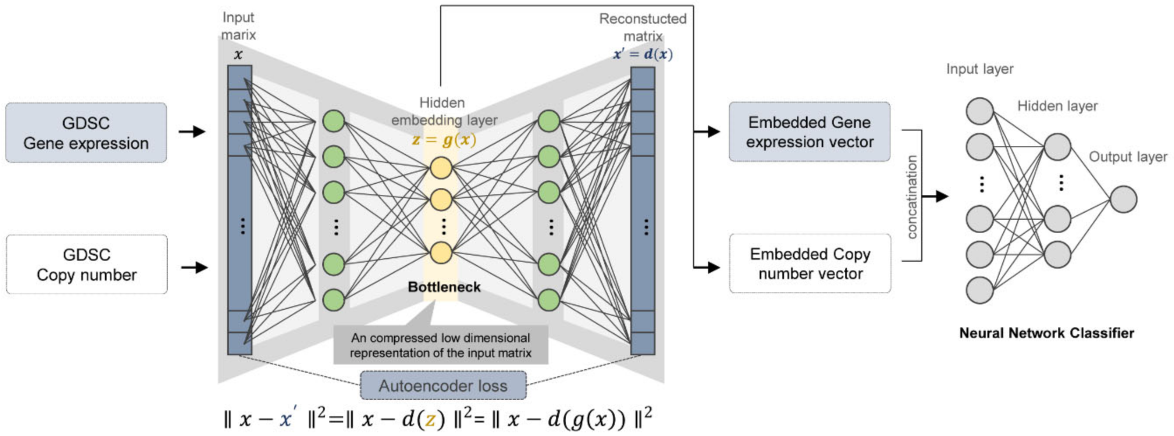

S1 Figure. Model architecture for predicting cell line response to drugs. The prediction model consists of an Autoencoder and a neural network (AE-NN). The autoencoder reduces high-dimensional omics data to low-dimensional data. Gene expression and copy number data are each passed through the autoencoder, and when the loss between the reconstructed data and the input is small enough, the hidden embedding layer (bottleneck) values of the two omics data are concatenated. The concatenated embedded matrix is passed through a neural network classifier, where the prediction probability is calculated and can be classified as 'Resistant' or 'Sensitive' based on a threshold. The predictive model is trained and tested with the GDSC gene expression and copy number data with five-fold cross-validation, and used to predict drug response in the sarcoma cell lines RD and SJCRH30.
