# Supplementary material for: Integrated drug response prediction models pinpoint repurposed drugs with effectiveness against rhabdomyosarcoma
Source: PLoS One. 2024 Jan 26;19(1):e0295629. doi: 10.1371/journal.pone.0295629 (PMC10817174; doi:10.1371/journal.pone.0295629)
Supplement: S1 Table — (PDF) [file pone.0295629.s003.pdf]

Table S1: List of experimented drugs and the number of GDSC samples

Drug	# Sample	# Resistant	# Sensitive	Ratio of minor
681640	759	689	70	0.09
17-AAG	850	801	49	0.06
5-Fluorouracil	916	822	94	0.10
A-443654	401	370	31	0.08
A-770041	402	238	164	0.41
ABT-263	849	626	223	0.26
ABT-869	924	813	111	0.12
ABT-888	850	752	98	0.12
AC220	923	776	147	0.16
AG-014699	921	841	80	0.09
AICAR	840	751	89	0.11
AKT-inhibitor-VIII	881	792	89	0.10
AMG-706	849	746	103	0.12
AP-24534	884	797	87	0.10
AR-42	913	832	81	0.09
AS601245	872	794	78	0.09
AS605240	921	807	114	0.12
AT-7519	922	865	57	0.06
ATRA	840	741	99	0.12
AUY922	866	815	51	0.06
AV-951	922	809	113	0.12
Axitinib	847	774	73	0.09
AZ628	404	339	65	0.16
AZD-0530	409	345	64	0.16
AZD7762	849	785	64	0.08
AZD8055	841	747	94	0.11
BAY-61-3606	872	802	70	0.08
Bexarotene	863	793	70	0.08
BI-2536	400	369	31	0.08
BIRB-0796	838	729	109	0.13
BIX02189	922	817	105	0.11
BMN-673	915	809	106	0.12
BMS-345541	926	806	120	0.13
BMS-509744	402	363	39	0.10
BMS-754807	871	754	117	0.13
Bortezomib	402	371	31	0.08
Bosutinib	851	772	79	0.09
Bryostatin-1	870	765	105	0.12
BX-795	849	773	76	0.09
BX-912	924	799	125	0.14
CAL-101	925	806	119	0.13
Camptothecin	849	790	59	0.07
CAY10603	916	833	83	0.09
CCT007093	921	810	111	0.12
CCT018159	897	837	60	0.07
CEP-701	849	768	81	0.10
Cetuximab	873	749	124	0.14
CGP-082996	401	372	29	0.07
CGP-60474	401	370	31	0.08
CH5424802	921	754	167	0.18
CI-1040	839	724	115	0.14
Cisplatin	850	771	79	0.09
CMK	401	376	25	0.06
CP466722	926	841	85	0.09
CP724714	924	813	111	0.12
Crizotinib	408	355	53	0.13
CUDC-101	905	825	80	0.09
CX-5461	920	397	523	0.57
Cyclopamine	397	336	61	0.15
Cytarabine	846	761	85	0.10
Dabrafenib	873	728	145	0.17
Dasatinib	398	265	133	0.33
DMOG	881	806	75	0.09
Docetaxel	850	784	66	0.08

Doxorubicin	875	812	63	0.07
EHT-1864	922	837	85	0.09
EKB-569	923	806	117	0.13
Elesclomol	849	792	57	0.07
Embelin	876	815	61	0.07
Epothilone-B	875	816	59	0.07
Erlotinib	372	308	64	0.17
Etoposide	885	830	55	0.06
EX-527	919	813	106	0.12
FH535	872	792	80	0.09
FK866	901	891	10	0.01
FMK	795	682	113	0.14
FR-180204	922	803	119	0.13
FTI-277	884	777	107	0.12
Gefitinib	846	727	119	0.14
Gemcitabine	870	815	55	0.06
Genentech-Cpd-10	926	823	103	0.11
GNF-2	399	349	50	0.13
GSK1070916	897	737	160	0.18
GSK-1904529A	875	765	110	0.13
GSK2126458	924	829	95	0.10
GSK429286A	926	803	123	0.13
GSK-650394	866	792	74	0.09
GSK690693	922	597	325	0.35
GW-2580	924	808	116	0.13
GW-441756	847	728	119	0.14
GW843682X	402	386	16	0.04
HG-5-113-01	497	454	43	0.09
HG-5-88-01	496	437	59	0.12
HG-6-64-1	880	811	69	0.08
I-BET-151	920	751	169	0.18
Imatinib	409	358	51	0.12
IOX2	929	808	121	0.13
IPA-3	874	824	50	0.06
JNJ-26854165	918	841	77	0.08
JNK-9L	884	813	71	0.08
JNK-Inhibitor-VIII	847	751	96	0.11
JQ12	878	794	84	0.10
JW-7-24-1	924	797	127	0.14
JW-7-52-1	386	353	33	0.09
KIN001-055	922	821	101	0.11
KIN001-102	925	818	107	0.12
KIN001-135	403	365	38	0.09
KIN001-236	922	791	131	0.14
KIN001-244	921	823	98	0.11
KIN001-260	921	672	249	0.27
KIN001-266	922	838	84	0.09
KIN001-270	922	804	118	0.13
KU-55933	848	764	84	0.10
Lapatinib	398	337	61	0.15
LAQ824	876	791	85	0.10
Lenalidomide	851	745	106	0.12
LFM-A13	874	783	91	0.10
LY317615	924	824	100	0.11
Masitinib	923	835	88	0.10
Methotrexate	849	717	132	0.16
MG-132	401	374	27	0.07
Midostaurin	885	809	76	0.09
Mitomycin-C	876	805	71	0.08
MK-2206	825	729	96	0.12
MLN4924	703	655	48	0.07
MP470	917	788	129	0.14
MPS-1-IN-1	920	862	58	0.06
MS-275	402	377	25	0.06
NG-25	922	826	96	0.10
Nilotinib	803	687	116	0.14
NPK76-II-72-1	922	829	93	0.10
NSC-207895	916	854	62	0.07
NSC-87877	880	773	107	0.12

NU-7441	846	770	76	0.09
Nutlin-3a	849	612	237	0.28
NVP-BEZ235	842	775	67	0.08
NVP-BHG712	922	819	103	0.11
NVP-TAE684	408	371	37	0.09
Obatoclax-Mesylate	867	801	66	0.08
OSI-027	918	856	62	0.07
OSI-906	871	747	124	0.14
OSI-930	923	815	108	0.12
OSU-03012	874	792	82	0.09
Oxozeaenol	916	796	120	0.13
PAC-1	863	802	61	0.07
Paclitaxel	402	376	26	0.06
Parthenolide	403	363	40	0.10
Pazopanib	872	777	95	0.11
PD-0325901	737	596	141	0.19
PD-0332991	825	755	70	0.08
PD-173074	849	735	114	0.13
PF-4708671	908	823	85	0.09
PF-562271	866	795	71	0.08
PFI-1	931	843	88	0.09
PHA-665752	409	346	63	0.15
PHA-793887	924	822	102	0.11
Phenformin	914	814	100	0.11
PI-103	915	838	77	0.08
PIK-93	922	768	154	0.17
piperlongumine	928	840	88	0.09
PXD101	896	818	78	0.09
Pyrimethamine	402	366	36	0.09
QL-VIII-58	499	468	31	0.06
QL-X-138	911	824	87	0.10
QL-XI-92	924	584	340	0.37
QL-XII-47	923	870	53	0.06
QL-XII-61	475	332	143	0.30
QS11	875	807	68	0.08
Rapamycin	366	297	69	0.19
RO-3306	849	742	107	0.13
Roscovitine	398	363	35	0.09
rTRAIL	920	663	257	0.28
Ruxolitinib	925	800	125	0.14
Salubrinal	399	371	28	0.07
SB-216763	746	654	92	0.12
SB-505124	928	824	104	0.11
SB52334	921	809	112	0.12
SB590885	726	619	107	0.15
SB-715992	925	891	34	0.04
SGC0946	910	821	89	0.10
Shikonin	881	798	83	0.09
SL-0101-1	834	748	86	0.10
SN-38	930	861	69	0.07
SNX-2112	914	874	40	0.04
Sorafenib	404	373	31	0.08
STF-62247	920	762	158	0.17
S-Trityl-L-cysteine	400	383	17	0.04
Sunitinib	401	375	26	0.06
T0901317	917	799	118	0.13
TAK-715	927	857	70	0.08
Tamoxifen	928	820	108	0.12
Temozolomide	910	807	103	0.11
Temsirolimus	837	721	116	0.14
TG101348	924	808	116	0.13
TGX221	401	345	56	0.14
Thapsigargin	860	803	57	0.07
THZ-2-102-1	905	826	79	0.09
THZ-2-49	919	881	38	0.04
Tipifarnib	873	772	101	0.12
TL-1-85	922	839	83	0.09
TL-2-105	927	800	127	0.14
TPCA-1	922	809	113	0.12

Trametinib	896	612	284	0.32
Tubastatin-A	919	611	308	0.34
TW-37	920	849	71	0.08
UNC1215	911	814	97	0.11
Vinblastine	850	786	64	0.08
Vinorelbine	885	833	52	0.06
Vismodegib	850	749	101	0.12
VNLG-124	919	785	134	0.15
Vorinostat	851	789	62	0.07
VX-11e	922	806	116	0.13
VX-680	396	313	83	0.21
VX-702	847	741	106	0.13
WH-4-023	399	313	86	0.22
WZ-1-84	401	347	54	0.13
WZ3105	924	856	68	0.07
XAV-939	919	819	100	0.11
XL-184	922	837	85	0.09
XL-880	919	840	79	0.09
XMD11-85h	497	451	46	0.09
XMD13-2	923	830	93	0.10
XMD14-99	924	764	160	0.17
XMD15-27	924	790	134	0.15
XMD8-85	398	362	36	0.09
XMD8-92	498	460	38	0.08
Y-39983	922	556	366	0.40
YK-4-279	794	736	58	0.07
YM155	891	861	30	0.03
YM201636	922	847	75	0.08
ZG-10	497	458	39	0.08
Zibotentan	922	823	99	0.11
Z-LLNle-CHO	401	371	30	0.07
ZM-447439	799	720	79	0.10
ZSTK474	925	820	105	0.11
**Avg.**	**794.9**	**701.5**	**93.4**	**0.1**
