# Supplementary material for: Integrated drug response prediction models pinpoint repurposed drugs with effectiveness against rhabdomyosarcoma
Source: PLoS One. 2024 Jan 26;19(1):e0295629. doi: 10.1371/journal.pone.0295629 (PMC10817174; doi:10.1371/journal.pone.0295629)
Supplement: S2 Table — Pred_RD and Pred_SJCRH30 are the predicted values of the RD and SJCRH30 cell lines mapped to probabilities using the sigmoid function of AE-NN. The predicted probabilities of each cell line were binarized based on the threshold in the last column of the table and inserted into the Response_RD and Response_SJCRH30 columns, respectively. The averaged predictive performance of the GDSC 5-fold cross-validation of AE-NN for each drug was evaluated with AUC and F1 scores, and each threshold was set as the value at the highest F1 score. Iorio_RD column is the published drug responses to RD cell line by Iorio et al. (2016). Of the 145 non-NA drugs with binarized drug responses in the Iorio_RD column, the responses of 140 drugs were consistent with the binarized prediction results of the AE-NN model (Response_RD column). The accuracy was 0.966, the sensitivity was 0.636, and the specificity was 0.993. Super.FELT_RD and Super.FELT_SJCRH30 are binarized prediction results by Super.FELT model of RD and SJCRH30 cell lines. The result of Super.FELT classifications of RD cell line were consistent with the Iorio’s for 57 of the 141 overlapped drugs. The accuracy was 0.404, the sensitivity was 0.8, and the specificity was 0.374. (PDF) [file pone.0295629.s004.pdf]

Table S2: Predicted drug response of RD and SJCRH30 cell lines

Pred_RD and Pred_SJCRH30 are the predicted values of the RD and SJCRH30 cell lines mapped to probabilities using the sigmoid function of AE-NN. The predicted probabilities of each cell line were binarized based on the threshold in the last column of the table and inserted into the Response_RD and Response_SJCRH30 columns, respectively. The averaged predictive performance of the GDSC 5-fold cross-validation of AE-NN for each drug was evaluated with AUC and F1 scores, and each threshold was set as the value at the highest F1 score. Iorio_RD column is the published drug responses to RD cell line by Iorio et al. (2016). Of the 145 non-NA drugs with binarized drug responses in the Iorio_RD column, the responses of 140 drugs were consistent with the binarized prediction results of the AE-NN model (Response_RD column). The accuracy was 0.966, the sensitivity was 0.636, and the specificity was 0.993. Super.FELT_RD and Super.FELT_SJCRH30 are binarized prediction results by Super.FELT model of RD and SJCRH30 cell lines. The result of Super.FELT classifications of RD cell line were consistent with the Iorio's for 57 of the 141 overlapped drugs. The accuracy was 0.404, the sensitivity was 0.8, and the specificity was 0.374.

Drug	Pred_RD	Pred _SJCRH30	Response _RD	Response _SJCRH30	Iorio_RD	Iorio _SJCRH30	Super.FELT _RD	Super.FELT _SJCRH30	AUC	F1	Threshold
AT-7519	0.03	0.08	R	R	R	R	S	S	0.90	0.54	0.44
CAY10603	0.07	0.25	R	R	R	R	S	S	0.89	0.55	0.39
WZ3105	0.07	0.08	R	R	R	R	S	S	0.88	0.55	0.44
TL-1-85	0.04	0.10	R	R	R	R	S	S	0.88	0.62	0.45
Methotrexate	0.06	0.32	R	R	NA	R	R	R	0.87	0.66	0.46
THZ-2-102-1	0.04	0.06	R	R	R	R	S	S	0.87	0.51	0.33
AZ628	0.30	0.07	R	R	NA	NA	R	R	0.87	0.60	0.37
XMD14-99	0.07	0.39	R	S	R	R	S	S	0.86	0.68	0.36
AR-42	0.07	0.22	R	R	R	R	S	S	0.86	0.49	0.29
NPK76-II-72-1	0.06	0.35	R	R	R	R	S	S	0.86	0.56	0.39
BIX02189	0.03	0.21	R	R	R	R	R	R	0.86	0.64	0.48
TG101348	0.16	0.13	R	R	R	R	R	R	0.86	0.63	0.34
Nutlin-3a	0.06	0.21	R	R	NA	R	S	R	0.86	0.71	0.48
KIN001-236	0.10	0.18	R	R	R	R	S	S	0.86	0.61	0.55
TPCA-1	0.06	0.07	R	R	R	R	S	S	0.85	0.60	0.37
5-Fluorouracil	0.04	0.05	R	R	R	R	R	R	0.85	0.58	0.50
NVP-BHG712	0.13	0.05	R	R	R	R	R	S	0.85	0.58	0.27
T0901317	0.05	0.09	R	R	R	R	S	S	0.85	0.64	0.54
TAK-715	0.08	0.06	R	R	R	R	S	S	0.85	0.48	0.40
NG-25	0.03	0.32	R	R	R	R	S	S	0.85	0.65	0.45
PHA-793887	0.06	0.11	R	R	R	R	S	R	0.85	0.61	0.42
KIN001-270	0.03	0.23	R	R	R	R	S	S	0.85	0.57	0.28
PI-103	0.04	0.11	R	R	R	R	S	S	0.85	0.55	0.44
YM201636	0.05	0.09	R	R	R	R	R	S	0.84	0.56	0.45
CP466722	0.03	0.09	R	R	R	R	S	S	0.84	0.58	0.51
JW-7-24-1	0.10	0.16	R	R	R	R	S	S	0.84	0.61	0.43
BX-912	0.06	0.41	R	R	R	R	S	S	0.84	0.59	0.44
KIN001-102	0.08	0.20	R	R	R	R	S	S	0.84	0.54	0.39
CUDC-101	0.05	0.33	R	R	R	R	S	S	0.84	0.51	0.50
Dabrafenib	0.13	0.06	R	R	R	R	R	R	0.84	0.58	0.46
KIN001-260	0.38	0.53	R	S	R	R	S	S	0.84	0.69	0.42
PIK-93	0.05	0.19	R	R	R	R	S	S	0.84	0.69	0.49
Zibotentan	0.03	0.19	R	R	R	R	R	R	0.84	0.55	0.47
ABT-263	0.49	0.85	R	S	NA	R	S	S	0.84	0.70	0.51
Trametinib	0.39	0.19	S	R	S	R	S	S	0.83	0.68	0.38
CI-1040	0.19	0.19	R	R	NA	R	R	R	0.83	0.53	0.30
AV-951	0.06	0.23	R	R	R	R	R	S	0.83	0.54	0.40
ZSTK474	0.07	0.13	R	R	R	R	S	S	0.83	0.58	0.52
Gefitinib	0.06	0.26	R	R	NA	R	R	R	0.83	0.61	0.46
SNX-2112	0.03	0.10	R	R	R	R	R	R	0.83	0.42	0.54
I-BET-151	0.16	0.23	R	R	R	R	-	-	0.83	0.68	0.49
XMD13-2	0.11	0.14	R	R	R	R	S	S	0.82	0.51	0.34
GSK1070916	0.08	0.43	R	S	R	R	S	S	0.82	0.58	0.33
Vorinostat	0.08	0.53	R	S	NA	S	S	S	0.82	0.44	0.38
OSI-027	0.04	0.06	R	R	R	R	S	S	0.82	0.43	0.40
Oxozeaenol	0.34	0.06	R	R	S	R	-	-	0.81	0.52	0.39
GSK429286A	0.07	0.15	R	R	R	R	S	S	0.81	0.52	0.51
WZ-1-84	0.11	0.15	R	R	NA	NA	R	R	0.81	0.53	0.47
VNLG-124	0.08	0.18	R	R	R	R	-	-	0.81	0.61	0.36
IPA-3	0.04	0.15	R	R	R	R	R	R	0.80	0.44	0.45
AC220	0.12	0.27	R	R	R	R	S	S	0.80	0.53	0.29
PD-0325901	0.19	0.13	R	R	NA	R	S	R	0.80	0.54	0.33
OSI-930	0.07	0.08	R	R	R	R	R	R	0.80	0.52	0.29
PXD101	0.11	0.27	R	S	R	R	S	S	0.80	0.44	0.24
Temozolomide	0.24	0.22	R	R	R	R	R	R	0.80	0.49	0.36
AZD8055	0.02	0.22	R	R	NA	R	S	S	0.80	0.48	0.30
STF-62247	0.07	0.24	R	R	R	R	S	S	0.79	0.59	0.47
AICAR	0.06	0.27	R	R	NA	R	S	S	0.79	0.49	0.34
Dasatinib	0.19	0.11	R	R	NA	NA	R	R	0.79	0.66	0.42
QL-X-138	0.05	0.10	R	R	R	R	S	R	0.79	0.48	0.37
Docetaxel	0.07	0.07	R	R	NA	R	R	R	0.79	0.38	0.41
EKB-569	0.06	0.19	R	R	R	R	R	R	0.79	0.50	0.31
XMD15-27	0.02	0.64	R	S	R	S	S	S	0.79	0.58	0.46
GSK690693	0.33	0.90	R	S	R	S	S	S	0.79	0.67	0.35
MPS-1-IN-1	0.03	0.10	R	R	R	R	S	S	0.78	0.40	0.32
TL-2-105	0.14	0.36	R	R	R	R	S	S	0.78	0.51	0.48
KIN001-244	0.05	0.17	R	R	R	R	S	S	0.78	0.49	0.38
THZ-2-49	0.04	0.09	R	R	R	R	R	R	0.78	0.38	0.20

Genentech-Cpd-10	0.13	0.27	R	R	R	R	S	S	0.78	0.49	0.56
ZM-447439	0.06	0.23	R	R	NA	R	S	S	0.77	0.46	0.50
Bosutinib	0.29	0.10	R	R	NA	R	R	R	0.77	0.41	0.45
BMS-345541	0.10	0.30	R	R	R	R	S	S	0.77	0.48	0.41
FMK	0.10	0.21	R	R	R	R	S	R	0.77	0.53	0.39
Vinblastine	0.09	0.20	R	R	NA	R	R	R	0.77	0.36	0.30
VX-702	0.18	0.09	R	R	NA	R	R	R	0.77	0.49	0.39
Camptothecin	0.05	0.09	R	R	NA	R	S	S	0.77	0.41	0.42
BX-795	0.04	0.09	R	R	NA	R	R	R	0.77	0.41	0.48
Tubastatin-A	0.79	0.51	S	S	S	R	S	S	0.77	0.63	0.33
UNC1215	0.03	0.33	R	R	R	S	R	R	0.77	0.50	0.38
Cytarabine	0.09	0.23	R	R	NA	S	R	R	0.77	0.48	0.33
FR-180204	0.16	0.18	R	R	R	R	R	R	0.77	0.47	0.38
PAC-1	0.06	0.15	R	R	R	R	S	S	0.76	0.50	0.50
HG-6-64-1	0.04	0.08	R	R	R	R	S	R	0.76	0.37	0.31
Crizotinib	0.18	0.13	R	R	NA	NA	R	R	0.76	0.51	0.40
Cisplatin	0.19	0.08	R	R	NA	R	R	R	0.76	0.42	0.37
WH-4-023	0.12	0.11	R	R	NA	NA	R	R	0.76	0.53	0.27
BMN-673	0.02	0.33	R	R	R	S	S	R	0.76	0.42	0.38
ATRA	0.11	0.18	R	R	NA	R	R	R	0.76	0.45	0.43
Y-39983	0.32	0.68	R	S	R	R	S	S	0.76	0.66	0.39
SB590885	0.38	0.16	S	R	NA	R	S	S	0.76	0.50	0.33
QL-XII-61	0.21	0.52	R	S	NA	R	S	S	0.75	0.62	0.41
Ruxolitinib	0.24	0.25	R	R	R	R	S	S	0.75	0.50	0.49
EX-527	0.08	0.13	R	R	R	R	R	S	0.75	0.40	0.28
XL-880	0.06	0.14	R	R	R	R	S	R	0.75	0.41	0.32
SN-38	0.02	0.17	R	R	R	R	R	R	0.75	0.45	0.34
XL-184	0.06	0.18	R	R	R	R	R	R	0.75	0.43	0.45
Etoposide	0.04	0.14	R	R	R	R	R	R	0.75	0.40	0.52
Lapatinib	0.21	0.11	R	R	NA	NA	R	R	0.75	0.55	0.52
CAL-101	0.16	0.19	R	R	R	R	S	S	0.75	0.54	0.49
MK-2206	0.05	0.17	R	R	NA	R	R	R	0.75	0.43	0.37
CEP-701	0.19	0.12	R	R	NA	R	R	R	0.75	0.40	0.45
Vismodegib	0.16	0.11	R	R	NA	R	R	R	0.75	0.43	0.40
CH5424802	0.12	0.73	R	S	R	S	S	S	0.75	0.50	0.31
AS605240	0.26	0.16	R	R	R	R	R	R	0.74	0.48	0.31
DMOG	0.04	0.10	R	R	R	R	S	S	0.74	0.38	0.35
QL-XI-92	0.60	0.74	S	S	S	S	S	S	0.74	0.63	0.35
GSK2126458	0.08	0.23	R	R	R	R	S	S	0.74	0.43	0.46
Phenformin	0.09	0.19	R	R	R	R	S	S	0.74	0.40	0.29
MLN4924	0.09	0.19	R	R	R	R	-	-	0.74	0.39	0.31
Nilotinib	0.19	0.25	R	R	NA	R	R	R	0.74	0.45	0.37
AP-24534	0.06	0.12	R	R	R	R	S	S	0.74	0.44	0.34
Embelin	0.03	0.10	R	R	R	R	R	R	0.74	0.33	0.26
AZD-0530	0.21	0.12	R	R	NA	NA	R	R	0.74	0.46	0.29
HG-5-113-01	0.03	0.11	R	R	NA	R	S	S	0.74	0.36	0.34
VX-11e	0.62	0.14	S	R	S	R	S	S	0.74	0.41	0.40
Lenalidomide	0.12	0.25	R	R	NA	R	R	R	0.74	0.45	0.54
MS-275	0.17	0.19	R	R	NA	NA	R	R	0.73	0.33	0.30
TGX221	0.12	0.06	R	R	NA	NA	R	R	0.73	0.47	0.29
OSI-906	0.19	0.64	R	S	S	S	S	S	0.73	0.42	0.26
BI-2536	0.14	0.16	R	R	NA	NA	R	R	0.73	0.39	0.23
XAV-939	0.08	0.07	R	R	R	R	S	S	0.73	0.38	0.44
GW-2580	0.26	0.50	R	S	R	R	S	S	0.73	0.40	0.36
PD-0332991	0.04	0.10	R	R	NA	R	R	R	0.73	0.38	0.34
AZD7762	0.02	0.17	R	R	NA	S	R	R	0.73	0.36	0.25
Tamoxifen	0.16	0.36	R	R	R	R	S	S	0.73	0.39	0.42
ABT-869	0.08	0.14	R	R	R	R	R	R	0.73	0.44	0.44
Mitomycin-C	0.04	0.19	R	R	R	R	R	R	0.73	0.37	0.26
SB52334	0.15	0.19	R	R	R	R	S	S	0.73	0.45	0.44
A-770041	0.38	0.32	S	R	NA	NA	R	R	0.73	0.67	0.34
Masitinib	0.13	0.07	R	R	R	R	R	R	0.72	0.41	0.40
KIN001-055	0.07	0.21	R	R	R	R	S	S	0.72	0.35	0.27
Temsirolimus	0.08	0.07	R	R	NA	R	R	R	0.72	0.40	0.38
Axitinib	0.03	0.16	R	R	NA	R	R	R	0.72	0.40	0.42
NU-7441	0.02	0.17	R	R	NA	R	R	R	0.72	0.36	0.31
SB-715992	0.02	0.05	R	R	R	R	R	R	0.72	0.21	0.29
AG-014699	0.04	0.05	R	R	R	R	R	R	0.72	0.35	0.42
QL-VIII-58	0.05	0.09	R	R	NA	R	R	R	0.72	0.32	0.28
Cetuximab	0.13	0.12	R	R	R	R	R	R	0.72	0.40	0.23
piperlongumine	0.09	0.10	R	R	S	R	R	R	0.72	0.32	0.23
JW-7-52-1	0.07	0.10	R	R	NA	NA	S	S	0.71	0.36	0.34
CMK	0.08	0.14	R	R	NA	NA	R	R	0.71	0.36	0.41
Gemcitabine	0.08	0.05	R	R	R	R	S	S	0.71	0.29	0.31
KU-55933	0.06	0.35	R	R	NA	R	R	R	0.70	0.39	0.48
TW-37	0.13	0.06	R	R	R	R	R	R	0.70	0.33	0.37
Erlotinib	0.12	0.19	R	R	NA	NA	R	R	0.70	0.51	0.51
ABT-888	0.28	0.28	R	R	NA	R	R	R	0.70	0.38	0.44
Cyclopamine	0.12	0.24	R	R	NA	NA	R	R	0.70	0.45	0.31
CGP-082996	0.07	0.19	R	R	NA	NA	S	S	0.70	0.31	0.25
SB-505124	0.02	0.31	R	R	R	R	S	S	0.70	0.36	0.32
SGC0946	0.21	0.17	R	R	R	R	S	S	0.70	0.38	0.31
BAY-61-3606	0.03	0.06	R	R	R	R	R	R	0.70	0.38	0.37
Sorafenib	0.13	0.09	R	R	NA	NA	R	R	0.70	0.39	0.35
HG-5-88-01	0.08	0.42	R	S	NA	S	S	S	0.69	0.38	0.36

PD-173074	0.07	0.56	R	S	NA	R	R	R	0.69	0.40	0.46
EHT-1864	0.06	0.21	R	R	R	R	R	R	0.69	0.32	0.28
rTRAIL	0.78	0.29	S	R	S	R	R	R	0.69	0.53	0.31
CX-5461	0.89	0.67	S	S	S	R	S	S	0.69	0.74	0.11
MG-132	0.09	0.15	R	R	NA	NA	S	S	0.69	0.42	0.33
Pyrimethamine	0.18	0.21	R	S	NA	NA	S	S	0.69	0.35	0.21
NSC-207895	0.44	0.09	S	R	S	R	S	S	0.69	0.26	0.17
BMS-754807	0.24	0.50	R	S	S	S	S	S	0.68	0.39	0.39
JNJ-26854165	0.12	0.07	R	R	R	R	R	R	0.68	0.28	0.30
CGP-60474	0.07	0.08	R	R	NA	NA	R	R	0.68	0.37	0.35
Pazopanib	0.08	0.12	R	R	R	R	S	S	0.68	0.36	0.33
Obatoclax-Mesylate	0.11	0.18	R	R	R	R	R	R	0.68	0.35	0.29
Thapsigargin	0.10	0.04	R	R	R	R	R	R	0.68	0.26	0.20
BMS-509744	0.10	0.10	R	R	NA	NA	S	S	0.68	0.33	0.25
YK-4-279	0.07	0.27	R	S	R	R	S	S	0.68	0.26	0.19
Z-LLNle-CHO	0.11	0.17	R	R	NA	NA	R	R	0.68	0.34	0.30
PFI-1	0.12	0.09	R	R	R	R	S	S	0.67	0.33	0.28
17-AAG	0.06	0.13	R	R	NA	R	R	R	0.67	0.20	0.16
CP724714	0.12	0.46	R	S	R	R	S	S	0.67	0.32	0.23
Imatinib	0.08	0.24	R	R	NA	NA	R	R	0.67	0.39	0.29
LY317615	0.12	0.11	R	R	R	R	S	S	0.67	0.32	0.38
Sunitinib	0.09	0.09	R	R	NA	NA	R	R	0.66	0.46	0.41
LAQ824	0.32	0.08	R	R	R	R	S	S	0.66	0.34	0.44
Midostaurin	0.07	0.05	R	R	R	R	R	R	0.66	0.29	0.19
Bortezomib	0.15	0.11	R	R	NA	NA	R	R	0.66	0.31	0.34
VX-680	0.21	0.46	R	S	NA	NA	R	R	0.66	0.46	0.38
NVP-TAE684	0.13	0.04	R	R	NA	NA	R	R	0.66	0.33	0.39
Epothilone-B	0.03	0.13	R	R	R	R	R	R	0.65	0.24	0.20
S-Trityl-L-cysteine	0.07	0.05	R	R	NA	NA	R	R	0.65	0.27	0.34
FH535	0.13	0.15	R	R	R	R	R	R	0.65	0.31	0.35
PHA-665752	0.15	0.10	R	R	NA	NA	R	R	0.64	0.38	0.26
Paclitaxel	0.09	0.12	R	R	NA	NA	R	R	0.64	0.25	0.28
CCT018159	0.05	0.08	R	R	R	R	R	R	0.64	0.27	0.27
OSU-03012	0.08	0.44	R	S	R	R	S	S	0.64	0.27	0.18
Elesclomol	0.08	0.10	R	R	NA	R	R	R	0.64	0.22	0.22
Salubrinal	0.12	0.23	R	R	NA	NA	S	S	0.63	0.31	0.37
RO-3306	0.17	0.13	R	R	NA	R	R	R	0.63	0.33	0.37
Parthenolide	0.07	0.19	R	R	NA	NA	S	S	0.63	0.33	0.42
ZG-10	0.06	0.16	R	R	NA	R	R	R	0.63	0.31	0.38
Roscovitine	0.08	0.06	R	R	NA	NA	R	R	0.63	0.34	0.27
GSK-650394	0.06	0.28	R	S	R	R	S	S	0.63	0.24	0.14
QL-XII-47	0.10	0.13	R	R	R	R	S	S	0.63	0.22	0.29
681640	0.13	0.17	R	R	NA	R	-	-	0.63	0.28	0.27
AMG-706	0.12	0.09	R	R	NA	R	S	S	0.62	0.30	0.29
XMD11-85h	0.10	0.15	R	R	NA	R	R	R	0.62	0.43	0.41
GNF-2	0.26	0.23	R	R	NA	NA	R	R	0.62	0.33	0.27
Rapamycin	0.19	0.27	R	R	NA	NA	S	R	0.62	0.42	0.28
XMD8-85	0.09	0.13	R	R	NA	NA	S	S	0.62	0.29	0.21
Bexarotene	0.06	0.10	R	R	R	R	R	R	0.62	0.25	0.26
IOX2	0.05	0.15	R	R	R	R	S	S	0.61	0.32	0.26
SB-216763	0.29	0.12	R	R	NA	R	R	R	0.61	0.32	0.30
FTI-277	0.08	0.16	R	R	R	R	R	R	0.61	0.31	0.25
JQ12	0.08	0.23	R	R	R	R	S	S	0.61	0.27	0.27
AUY922	0.04	0.12	R	R	R	R	R	R	0.61	0.19	0.23
Doxorubicin	0.04	0.10	R	R	R	R	R	R	0.61	0.26	0.28
MP470	0.13	0.51	R	S	R	R	S	S	0.61	0.33	0.24
Shikonin	0.08	0.10	R	R	R	R	R	R	0.61	0.26	0.22
PF-4708671	0.08	0.15	R	R	R	R	R	R	0.60	0.28	0.22
AKT-inhibitor-VIII	0.07	0.09	R	R	R	R	R	R	0.60	0.26	0.25
JNK-Inhibitor-VIII	0.33	0.09	S	R	NA	R	R	R	0.59	0.26	0.25
KIN001-135	0.07	0.42	R	S	NA	NA	R	R	0.59	0.28	0.20
A-443654	0.08	0.09	R	R	NA	NA	R	R	0.58	0.28	0.19
Bryostatin-1	0.11	0.26	R	S	R	R	S	S	0.58	0.28	0.19
BIRB-0796	0.08	0.15	R	R	NA	R	S	S	0.58	0.29	0.16
YM155	0.07	0.04	R	R	R	R	R	S	0.58	0.18	0.17
GW-441756	0.27	0.31	S	S	NA	S	S	R	0.58	0.31	0.20
NSC-87877	0.10	0.19	R	S	R	R	R	R	0.57	0.27	0.18
FK866	0.02	0.08	R	S	R	R	S	S	0.57	0.07	0.08
CCT007093	0.23	0.40	R	S	R	R	R	R	0.57	0.27	0.29
SL-0101-1	0.15	0.26	R	R	NA	R	R	R	0.57	0.25	0.39
GW843682X	0.10	0.15	R	R	NA	NA	R	R	0.57	0.23	0.21
LFM-A13	0.05	0.23	R	R	R	R	S	S	0.57	0.30	0.39
PF-562271	0.07	0.11	R	R	R	R	S	S	0.56	0.22	0.17
QS11	0.06	0.16	R	R	R	R	R	R	0.56	0.22	0.22
NVP-BEZ235	0.06	0.19	R	R	NA	R	R	R	0.56	0.21	0.25
XMD8-92	0.08	0.22	R	R	NA	R	S	S	0.55	0.26	0.32
KIN001-266	0.17	0.27	R	S	R	R	S	S	0.54	0.24	0.21
Tipifarnib	0.06	0.28	R	S	R	R	S	S	0.53	0.25	0.23
JNK-9L	0.08	0.09	R	R	R	R	S	R	0.52	0.19	0.22
Vinorelbine	0.02	0.20	R	S	R	R	S	S	0.52	0.15	0.12
AS601245	0.08	0.16	R	R	R	R	S	S	0.52	0.21	0.18
GSK-1904529A	0.21	0.36	S	S	R	R	S	S	0.48	0.23	0.07
**Avg.**									**0.72**	**0.42**	
