# Supplementary material for: Integrated drug response prediction models pinpoint repurposed drugs with effectiveness against rhabdomyosarcoma
Source: PLoS One. 2024 Jan 26;19(1):e0295629. doi: 10.1371/journal.pone.0295629 (PMC10817174; doi:10.1371/journal.pone.0295629)
Supplement: S3 Table — (PDF) [file pone.0295629.s005.pdf]

## Table S3: Confusion matrix

| | | (Iorio et al., 2016) | | | |
|---|---|---|---|---|---|
| | | RD | | SJCRH30 | |
| | | Sensitive | Resistant | Sensitive | Resistant |
| **AE-NN** | Sensitive | 7 (4.8%) | 1 (0.7%) | 9 (4.6%) | 24 (12.2%) |
| | Resistant | 4 (2.8%) | 133 (91.7%) | 4 (2.0%) | 159 (81.1%) |
| **Super.FELT** | Sensitive | 8 (5.7%) | 81 (57.4%) | 8 (4.1%) | 93 (47.4%) |
| | Resistant | 2 (1.4%) | 50 (35.5%) | 5 (2.6%) | 85 (43.4%) |
