# Supplementary material for: Integrated drug response prediction models pinpoint repurposed drugs with effectiveness against rhabdomyosarcoma
Source: PLoS One. 2024 Jan 26;19(1):e0295629. doi: 10.1371/journal.pone.0295629 (PMC10817174; doi:10.1371/journal.pone.0295629)
Supplement: S4 Table — (PDF) [file pone.0295629.s006.pdf]

Table S4: Predicted drug response of RD and SJCRH30 cell lines (w/o RD and SJCRH30 from training data)

Drug	Pred_RD	Pred_SJCRH30	Response_RD	Response_SJCRH30	AUC	F1	Threshold
AT-7519	0.02	0.06	R	R	0.90	0.58	0.47
CAY10603	0.07	0.27	R	R	0.87	0.57	0.55
WZ3105	0.06	0.06	R	R	0.86	0.53	0.36
TL-1-85	0.04	0.13	R	R	0.90	0.62	0.46
Methotrexate	0.06	0.27	R	R	0.88	0.64	0.55
THZ-2-102-1	0.04	0.07	R	R	0.85	0.52	0.33
AZ628	0.28	0.09	R	R	0.85	0.60	0.36
XMD14-99	0.09	0.48	R	S	0.86	0.67	0.46
AR-42	0.08	0.19	R	R	0.85	0.48	0.27
NPK76-II-72-1	0.07	0.32	R	R	0.88	0.57	0.37
BIX02189	0.04	0.20	R	R	0.85	0.62	0.62
TG101348	0.21	0.13	R	R	0.86	0.65	0.54
Nutlin-3a	0.06	0.19	R	R	0.86	0.70	0.43
KIN001-236	0.09	0.16	R	R	0.85	0.62	0.51
TPCA-1	0.05	0.08	R	R	0.85	0.59	0.34
5-Fluorouracil	0.05	0.05	R	R	0.82	0.54	0.53
NVP-BHG712	0.11	0.04	R	R	0.86	0.59	0.41
T0901317	0.08	0.13	R	R	0.85	0.66	0.49
TAK-715	0.07	0.06	R	R	0.86	0.49	0.41
NG-25	0.03	0.36	R	R	0.84	0.60	0.45
PHA-793887	0.08	0.16	R	R	0.86	0.60	0.34
KIN001-270	0.03	0.19	R	R	0.85	0.54	0.24
PI-103	0.03	0.07	R	R	0.84	0.56	0.41
YM201636	0.06	0.11	R	R	0.85	0.58	0.47
CP466722	0.04	0.08	R	R	0.86	0.62	0.47
JW-7-24-1	0.12	0.21	R	R	0.86	0.64	0.40
BX-912	0.05	0.36	R	R	0.81	0.56	0.44
KIN001-102	0.09	0.24	R	R	0.84	0.55	0.38
CUDC-101	0.06	0.32	R	R	0.86	0.51	0.38
Dabrafenib	0.14	0.08	R	R	0.84	0.57	0.33
KIN001-260	0.38	0.49	R	S	0.83	0.69	0.42
PIK-93	0.05	0.17	R	R	0.85	0.69	0.41
Zibotentan	0.03	0.15	R	R	0.83	0.55	0.40
ABT-263	0.47	0.85	R	S	0.84	0.67	0.51
Trametinib	0.35	0.15	S	R	0.85	0.70	0.34
CI-1040	0.18	0.13	R	R	0.86	0.55	0.39
AV-951	0.06	0.28	R	R	0.83	0.53	0.28
ZSTK474	0.10	0.12	R	R	0.81	0.58	0.44
Gefitinib	0.05	0.29	R	R	0.80	0.59	0.52
SNX-2112	0.01	0.05	R	R	0.86	0.46	0.33
I-BET-151	0.16	0.20	R	R	0.85	0.68	0.57
XMD13-2	0.13	0.18	R	R	0.82	0.53	0.37
GSK1070916	0.08	0.44	R	S	0.81	0.57	0.36
Vorinostat	0.08	0.51	R	S	0.83	0.46	0.39
OSI-027	0.05	0.05	R	R	0.83	0.42	0.38
Oxozeaenol	0.26	0.06	R	R	0.81	0.53	0.46
GSK429286A	0.07	0.08	R	R	0.79	0.48	0.28
WZ-1-84	0.13	0.21	R	R	0.78	0.49	0.34
VNLG-124	0.08	0.15	R	R	0.81	0.60	0.45
IPA-3	0.03	0.12	R	R	0.82	0.44	0.36
AC220	0.10	0.27	R	R	0.81	0.55	0.31
PD-0325901	0.20	0.16	R	R	0.80	0.56	0.32
OSI-930	0.09	0.13	R	R	0.80	0.54	0.40
PXD101	0.10	0.24	R	S	0.81	0.48	0.23
Temozolomide	0.18	0.18	R	R	0.77	0.49	0.46
AZD8055	0.02	0.22	R	R	0.79	0.49	0.30
STF-62247	0.06	0.25	R	R	0.79	0.60	0.55
AICAR	0.07	0.24	R	R	0.79	0.49	0.51
Dasatinib	0.20	0.11	R	R	0.82	0.70	0.41
QL-X-138	0.08	0.12	R	R	0.81	0.49	0.41
Docetaxel	0.08	0.06	R	R	0.80	0.38	0.36
EKB-569	0.09	0.18	R	R	0.80	0.51	0.34
XMD15-27	0.03	0.68	R	S	0.76	0.57	0.52
GSK690693	0.33	0.90	R	S	0.80	0.68	0.39
MPS-1-IN-1	0.05	0.13	R	R	0.75	0.42	0.37
TL-2-105	0.15	0.28	R	R	0.76	0.53	0.52
KIN001-244	0.04	0.19	R	R	0.77	0.49	0.44
THZ-2-49	0.04	0.05	R	R	0.76	0.34	0.26
Genentech-Cpd-10	0.15	0.21	R	R	0.77	0.52	0.44

ZM-447439	0.06	0.17	R	R	0.80	0.47	0.50
Bosutinib	0.36	0.10	R	R	0.78	0.42	0.45
BMS-345541	0.08	0.22	R	R	0.78	0.49	0.31
FMK	0.08	0.23	R	R	0.77	0.55	0.39
Vinblastine	0.05	0.10	R	R	0.77	0.37	0.29
VX-702	0.15	0.11	R	R	0.77	0.48	0.35
Camptothecin	0.07	0.08	R	R	0.81	0.42	0.32
BX-795	0.03	0.09	R	R	0.76	0.41	0.28
Tubastatin-A	0.79	0.57	S	S	0.79	0.65	0.39
UNC1215	0.02	0.24	R	R	0.77	0.47	0.33
Cytarabine	0.10	0.29	R	R	0.77	0.44	0.35
FR-180204	0.24	0.17	R	R	0.75	0.48	0.52
PAC-1	0.04	0.14	R	R	0.78	0.54	0.64
HG-6-64-1	0.06	0.09	R	R	0.75	0.37	0.26
Crizotinib	0.17	0.14	R	R	0.73	0.47	0.51
Cisplatin	0.18	0.04	R	R	0.73	0.37	0.22
WH-4-023	0.14	0.12	R	R	0.75	0.53	0.40
BMN-673	0.03	0.38	R	R	0.74	0.42	0.38
ATRA	0.11	0.21	R	R	0.71	0.39	0.38
Y-39983	0.31	0.70	R	S	0.76	0.66	0.33
SB590885	0.34	0.18	S	R	0.74	0.49	0.33
QL-XII-61	0.22	0.57	R	S	0.74	0.61	0.44
Ruxolitinib	0.30	0.33	R	R	0.75	0.50	0.49
EX-527	0.11	0.21	R	R	0.75	0.41	0.35
XL-880	0.06	0.09	R	R	0.76	0.39	0.33
SN-38	0.02	0.16	R	R	0.78	0.48	0.40
XL-184	0.07	0.14	R	R	0.75	0.44	0.38
Etoposide	0.05	0.10	R	R	0.73	0.38	0.52
Lapatinib	0.22	0.13	R	R	0.75	0.52	0.44
CAL-101	0.10	0.26	R	R	0.74	0.53	0.52
MK-2206	0.04	0.18	R	R	0.77	0.44	0.32
CEP-701	0.16	0.12	R	R	0.76	0.40	0.43
Vismodegib	0.15	0.15	R	R	0.73	0.39	0.25
CH5424802	0.14	0.72	R	S	0.72	0.48	0.41
AS605240	0.27	0.12	R	R	0.76	0.47	0.37
DMOG	0.05	0.08	R	R	0.73	0.34	0.27
QL-XI-92	0.64	0.77	S	S	0.76	0.64	0.31
GSK2126458	0.05	0.22	R	R	0.77	0.47	0.52
Phenformin	0.10	0.17	R	R	0.75	0.41	0.47
MLN4924	0.11	0.23	R	R	0.75	0.40	0.31
Nilotinib	0.12	0.23	R	R	0.75	0.45	0.29
AP-24534	0.05	0.07	R	R	0.75	0.44	0.31
Embelin	0.03	0.10	R	R	0.73	0.34	0.28
AZD-0530	0.20	0.12	R	R	0.72	0.44	0.26
HG-5-113-01	0.03	0.12	R	R	0.76	0.40	0.30
VX-11e	0.61	0.11	S	R	0.73	0.42	0.23
Lenalidomide	0.09	0.16	R	R	0.73	0.48	0.51
MS-275	0.15	0.17	R	R	0.71	0.34	0.29
TGX221	0.11	0.07	R	R	0.68	0.39	0.38
OSI-906	0.18	0.62	R	S	0.72	0.42	0.34
BI-2536	0.10	0.16	R	R	0.77	0.45	0.34
XAV-939	0.08	0.06	R	R	0.71	0.36	0.29
GW-2580	0.31	0.52	R	S	0.74	0.40	0.34
PD-0332991	0.04	0.10	R	R	0.71	0.35	0.27
AZD7762	0.03	0.21	R	R	0.76	0.41	0.44
Tamoxifen	0.10	0.31	R	R	0.70	0.35	0.32
ABT-869	0.06	0.16	R	R	0.73	0.45	0.46
Mitomycin-C	0.05	0.21	R	R	0.71	0.35	0.37
SB52334	0.13	0.19	R	R	0.75	0.42	0.31
A-770041	0.35	0.31	S	R	0.72	0.68	0.33
Masitinib	0.17	0.07	R	R	0.74	0.39	0.28
KIN001-055	0.08	0.20	R	R	0.75	0.36	0.26
Temsirolimus	0.08	0.06	R	R	0.71	0.39	0.41
Axitinib	0.04	0.20	R	R	0.75	0.37	0.44
NU-7441	0.02	0.14	R	R	0.74	0.38	0.41
SB-715992	0.02	0.06	R	R	0.74	0.25	0.25
AG-014699	0.04	0.05	R	R	0.69	0.35	0.26
QL-VIII-58	0.06	0.08	R	R	0.62	0.28	0.33
Cetuximab	0.10	0.11	R	R	0.72	0.44	0.39
piperlongumine	0.10	0.10	R	R	0.70	0.30	0.25
JW-7-52-1	0.09	0.12	R	R	0.66	0.29	0.30
CMK	0.08	0.14	R	R	0.68	0.35	0.33
Gemcitabine	0.08	0.05	R	R	0.70	0.26	0.25
KU-55933	0.03	0.36	R	R	0.69	0.37	0.48
TW-37	0.15	0.06	R	R	0.70	0.34	0.25
Erlotinib	0.11	0.16	R	R	0.67	0.50	0.55

ABT-888	0.21	0.16	R	R	0.67	0.33	0.36
Cyclopamine	0.15	0.24	R	R	0.72	0.45	0.36
CGP-082996	0.07	0.18	R	R	0.71	0.30	0.20
SB-505124	0.02	0.32	R	R	0.69	0.35	0.33
SGC0946	0.14	0.14	R	R	0.66	0.35	0.43
BAY-61-3606	0.05	0.06	R	R	0.69	0.35	0.31
Sorafenib	0.15	0.09	R	R	0.67	0.37	0.34
HG-5-88-01	0.04	0.39	R	S	0.73	0.42	0.28
PD-173074	0.05	0.60	R	S	0.72	0.41	0.33
EHT-1864	0.05	0.22	R	R	0.68	0.29	0.28
rTRAIL	0.88	0.26	S	R	0.70	0.54	0.33
CX-5461	0.86	0.64	S	S	0.71	0.75	0.20
MG-132	0.10	0.21	R	R	0.72	0.39	0.30
Pyrimethamine	0.16	0.21	R	S	0.60	0.30	0.21
NSC-207895	0.43	0.09	S	R	0.70	0.31	0.26
BMS-754807	0.24	0.57	R	S	0.67	0.37	0.28
JNJ-26854165	0.11	0.06	R	R	0.69	0.31	0.18
CGP-60474	0.09	0.11	R	R	0.65	0.38	0.40
Pazopanib	0.10	0.12	R	R	0.66	0.34	0.37
Obatoclax-Mesylate	0.11	0.19	R	R	0.71	0.35	0.25
Thapsigargin	0.10	0.04	R	R	0.67	0.26	0.24
BMS-509744	0.09	0.09	R	R	0.69	0.34	0.32
YK-4-279	0.10	0.30	R	S	0.65	0.27	0.22
Z-LLNle-CHO	0.12	0.19	R	R	0.65	0.27	0.24
PFI-1	0.08	0.09	R	R	0.71	0.35	0.24
17-AAG	0.08	0.10	R	R	0.71	0.26	0.22
CP724714	0.13	0.36	R	S	0.63	0.30	0.26
Imatinib	0.10	0.22	R	R	0.69	0.42	0.36
LY317615	0.11	0.09	R	R	0.68	0.33	0.23
Sunitinib	0.08	0.06	R	R	0.65	0.38	0.48
LAQ824	0.33	0.09	R	R	0.68	0.36	0.44
Midostaurin	0.07	0.05	R	R	0.66	0.29	0.20
Bortezomib	0.17	0.13	R	R	0.73	0.37	0.34
VX-680	0.25	0.45	R	S	0.68	0.48	0.40
NVP-TAE684	0.16	0.06	R	R	0.63	0.32	0.32
Epothilone-B	0.04	0.17	R	R	0.64	0.22	0.18
S-Trityl-L-cysteine	0.06	0.05	R	R	0.74	0.43	0.36
FH535	0.15	0.16	R	R	0.68	0.36	0.27
PHA-665752	0.17	0.11	R	R	0.60	0.34	0.27
Paclitaxel	0.10	0.11	R	R	0.63	0.30	0.31
CCT018159	0.06	0.08	R	R	0.65	0.26	0.24
OSU-03012	0.08	0.45	R	S	0.69	0.31	0.24
Elesclomol	0.09	0.08	R	R	0.66	0.24	0.23
Salubrinal	0.10	0.23	R	R	0.68	0.32	0.35
RO-3306	0.20	0.11	R	R	0.66	0.33	0.37
Parthenolide	0.06	0.17	R	R	0.65	0.36	0.36
ZG-10	0.06	0.14	R	R	0.66	0.31	0.20
Roscovitine	0.11	0.07	R	R	0.64	0.31	0.30
GSK-650394	0.07	0.31	R	S	0.62	0.23	0.15
QL-XII-47	0.10	0.12	R	R	0.66	0.24	0.23
681640	0.13	0.21	R	R	0.64	0.27	0.22
AMG-706	0.16	0.10	S	R	0.56	0.25	0.13
XMD11-85h	0.12	0.22	R	R	0.65	0.38	0.41
GNF-2	0.26	0.21	S	R	0.60	0.37	0.25
Rapamycin	0.14	0.26	R	R	0.62	0.42	0.28
XMD8-85	0.11	0.18	R	R	0.70	0.35	0.26
Bexarotene	0.06	0.10	R	R	0.66	0.28	0.20
IOX2	0.03	0.16	R	R	0.64	0.32	0.22
SB-216763	0.29	0.11	R	R	0.65	0.36	0.32
FTI-277	0.07	0.15	R	R	0.63	0.32	0.32
JQ12	0.07	0.19	R	R	0.62	0.29	0.26
AUY922	0.03	0.09	R	R	0.64	0.20	0.13
Doxorubicin	0.04	0.10	R	R	0.66	0.28	0.24
MP470	0.15	0.51	R	S	0.63	0.35	0.29
Shikonin	0.12	0.15	R	R	0.58	0.25	0.31
PF-4708671	0.10	0.09	R	R	0.65	0.28	0.26
AKT-inhibitor-VIII	0.12	0.12	R	R	0.60	0.27	0.21
JNK-Inhibitor-VIII	0.35	0.09	R	R	0.61	0.30	0.37
KIN001-135	0.06	0.39	R	S	0.63	0.36	0.37
A-443654	0.09	0.08	R	R	0.61	0.32	0.31
Bryostatin-1	0.10	0.23	R	S	0.63	0.31	0.23
BIRB-0796	0.12	0.17	R	R	0.57	0.28	0.18
YM155	0.09	0.03	R	R	0.57	0.23	0.33
GW-441756	0.23	0.32	S	S	0.56	0.30	0.19
NSC-87877	0.11	0.15	R	R	0.59	0.28	0.16
FK866	0.01	0.04	R	R	0.67	0.08	0.08

CCT007093	0.19	0.35	R	S	0.59	0.29	0.28
SL-0101-1	0.18	0.29	R	S	0.65	0.30	0.28
GW843682X	0.10	0.15	R	R	0.57	0.23	0.20
LFM-A13	0.04	0.18	R	R	0.60	0.32	0.41
PF-562271	0.09	0.11	R	R	0.60	0.26	0.34
QS11	0.05	0.20	R	R	0.59	0.23	0.20
NVP-BEZ235	0.07	0.18	R	R	0.59	0.23	0.22
XMD8-92	0.06	0.19	R	R	0.54	0.33	0.40
KIN001-266	0.21	0.29	S	S	0.56	0.22	0.15
Tipifarnib	0.09	0.28	R	S	0.54	0.23	0.14
JNK-9L	0.08	0.08	R	R	0.47	0.18	0.19
Vinorelbine	0.02	0.20	R	S	0.53	0.18	0.19
AS601245	0.08	0.16	R	R	0.58	0.25	0.22
GSK-1904529A	0.22	0.30	S	S	0.49	0.24	0.12
**Avg.**					**0.73**	**0.42**	
