# Supplementary material for: Integrated drug response prediction models pinpoint repurposed drugs with effectiveness against rhabdomyosarcoma
Source: PLoS One. 2024 Jan 26;19(1):e0295629. doi: 10.1371/journal.pone.0295629 (PMC10817174; doi:10.1371/journal.pone.0295629)
Supplement: S5 Table — (PDF) [file pone.0295629.s007.pdf]

Table S5: Predicted drug response of RD and SJCRH30 cell lines (best epoch result)

Drug	Pred_RD	Pred_SJCRH30	Response_RD	Response_SJCRH30	AUC	F1	Threshold
AT-7519	0.02	0.05	R	R	0.87	0.56	0.44
CAY10603	0.04	0.26	R	R	0.88	0.61	0.32
WZ3105	0.04	0.05	R	R	0.88	0.52	0.46
TL-1-85	0.02	0.09	R	R	0.88	0.61	0.59
Methotrexate	0.04	0.28	R	R	0.85	0.64	0.40
THZ-2-102-1	0.03	0.05	R	R	0.86	0.50	0.36
AZ628	0.23	0.05	R	R	0.85	0.60	0.37
XMD14-99	0.04	0.46	R	S	0.87	0.68	0.39
AR-42	0.07	0.22	R	R	0.84	0.49	0.36
NPK76-II-72-1	0.06	0.29	R	R	0.85	0.56	0.34
BIX02189	0.03	0.18	R	R	0.84	0.63	0.44
TG101348	0.12	0.10	R	R	0.85	0.63	0.32
Nutlin-3a	0.03	0.23	R	R	0.84	0.67	0.52
KIN001-236	0.06	0.10	R	R	0.85	0.60	0.53
TPCA-1	0.02	0.06	R	R	0.85	0.60	0.38
5-Fluorouracil	0.04	0.04	R	R	0.83	0.54	0.44
NVP-BHG712	0.09	0.03	R	R	0.87	0.58	0.40
T0901317	0.04	0.09	R	R	0.86	0.63	0.44
TAK-715	0.06	0.04	R	R	0.84	0.45	0.28
NG-25	0.02	0.32	R	R	0.83	0.62	0.46
PHA-793887	0.03	0.09	R	R	0.86	0.61	0.33
KIN001-270	0.01	0.21	R	R	0.86	0.57	0.24
PI-103	0.02	0.09	R	R	0.84	0.54	0.54
YM201636	0.03	0.05	R	R	0.85	0.59	0.48
CP466722	0.02	0.06	R	R	0.86	0.61	0.50
JW-7-24-1	0.08	0.17	R	R	0.86	0.62	0.50
BX-912	0.04	0.24	R	R	0.83	0.57	0.39
KIN001-102	0.06	0.19	R	R	0.82	0.52	0.35
CUDC-101	0.03	0.26	R	R	0.83	0.53	0.45
Dabrafenib	0.13	0.04	R	R	0.83	0.56	0.41
KIN001-260	0.40	0.48	R	S	0.81	0.67	0.42
PIK-93	0.04	0.14	R	R	0.83	0.69	0.45
Zibotentan	0.02	0.14	R	R	0.83	0.55	0.36
ABT-263	0.42	0.82	R	S	0.84	0.69	0.51
Trametinib	0.26	0.11	S	R	0.85	0.70	0.24
CI-1040	0.17	0.14	R	R	0.84	0.55	0.37
AV-951	0.04	0.16	R	R	0.83	0.54	0.28
ZSTK474	0.06	0.13	R	R	0.84	0.57	0.48
Gefitinib	0.03	0.21	R	R	0.81	0.60	0.39
SNX-2112	0.01	0.05	R	R	0.83	0.38	0.33
I-BET-151	0.22	0.20	R	R	0.82	0.67	0.57
XMD13-2	0.07	0.10	R	R	0.83	0.54	0.42
GSK1070916	0.06	0.44	R	S	0.83	0.58	0.34
Vorinostat	0.04	0.39	R	S	0.81	0.39	0.26
OSI-027	0.03	0.03	R	R	0.78	0.39	0.42
Oxozeaenol	0.27	0.04	R	R	0.82	0.53	0.33
GSK429286A	0.05	0.07	R	R	0.79	0.47	0.25
WZ-1-84	0.07	0.11	R	R	0.77	0.53	0.42
VNLG-124	0.05	0.16	R	R	0.80	0.62	0.44
IPA-3	0.03	0.10	R	R	0.78	0.44	0.44
AC220	0.07	0.32	R	R	0.82	0.54	0.34
PD-0325901	0.12	0.08	R	R	0.77	0.53	0.40
OSI-930	0.05	0.05	R	R	0.80	0.54	0.37
PXD101	0.11	0.27	R	S	0.81	0.48	0.24
Temozolomide	0.22	0.17	R	R	0.78	0.47	0.36
AZD8055	0.01	0.26	R	R	0.79	0.48	0.30
STF-62247	0.06	0.23	R	R	0.77	0.59	0.50
AICAR	0.05	0.27	R	R	0.78	0.46	0.47
Dasatinib	0.16	0.06	R	R	0.81	0.69	0.31
QL-X-138	0.05	0.09	R	R	0.77	0.47	0.40
Docetaxel	0.05	0.03	R	R	0.78	0.38	0.21
EKB-569	0.05	0.12	R	R	0.77	0.49	0.33
XMD15-27	0.01	0.75	R	S	0.80	0.58	0.44
GSK690693	0.34	0.92	R	S	0.79	0.67	0.35
MPS-1-IN-1	0.02	0.08	R	R	0.76	0.39	0.37
TL-2-105	0.11	0.26	R	R	0.77	0.52	0.40
KIN001-244	0.03	0.11	R	R	0.78	0.48	0.32
THZ-2-49	0.04	0.05	R	R	0.78	0.38	0.32
Genentech-Cpd-10	0.10	0.22	R	R	0.75	0.48	0.60
ZM-447439	0.03	0.14	R	R	0.78	0.48	0.42
Bosutinib	0.36	0.06	R	R	0.79	0.39	0.45

BMS-345541	0.07	0.25	R	R	0.76	0.50	0.37
FMK	0.07	0.19	R	R	0.74	0.53	0.34
Vinblastine	0.03	0.09	R	R	0.80	0.42	0.29
VX-702	0.16	0.05	R	R	0.78	0.49	0.41
Camptothecin	0.03	0.04	R	R	0.79	0.40	0.37
BX-795	0.03	0.07	R	R	0.71	0.36	0.57
Tubastatin-A	0.83	0.73	S	S	0.78	0.66	0.39
UNC1215	0.01	0.30	R	R	0.78	0.50	0.38
Cytarabine	0.07	0.23	R	R	0.80	0.46	0.27
FR-180204	0.19	0.13	R	R	0.78	0.46	0.33
PAC-1	0.05	0.12	R	R	0.79	0.53	0.41
HG-6-64-1	0.03	0.05	R	R	0.74	0.36	0.25
Crizotinib	0.15	0.14	R	R	0.76	0.51	0.37
Cisplatin	0.12	0.03	R	R	0.74	0.37	0.31
WH-4-023	0.08	0.06	R	R	0.77	0.54	0.41
BMN-673	0.01	0.29	R	R	0.73	0.42	0.33
ATRA	0.06	0.11	R	R	0.75	0.43	0.29
Y-39983	0.19	0.65	R	S	0.76	0.66	0.25
SB590885	0.38	0.12	S	R	0.73	0.47	0.33
QL-XII-61	0.18	0.63	R	S	0.73	0.59	0.38
Ruxolitinib	0.30	0.30	R	R	0.75	0.50	0.36
EX-527	0.08	0.14	R	R	0.73	0.42	0.25
XL-880	0.03	0.06	R	R	0.76	0.40	0.20
SN-38	0.02	0.13	R	R	0.78	0.45	0.45
XL-184	0.05	0.13	R	R	0.76	0.44	0.31
Etoposide	0.03	0.09	R	R	0.74	0.38	0.54
Lapatinib	0.17	0.10	R	R	0.74	0.53	0.45
CAL-101	0.12	0.17	R	R	0.75	0.53	0.54
MK-2206	0.02	0.13	R	R	0.76	0.46	0.36
CEP-701	0.13	0.07	R	R	0.74	0.41	0.40
Vismodegib	0.15	0.11	R	R	0.75	0.40	0.19
CH5424802	0.07	0.72	R	S	0.74	0.49	0.39
AS605240	0.27	0.08	R	R	0.74	0.45	0.35
DMOG	0.04	0.05	R	R	0.75	0.38	0.22
QL-XI-92	0.74	0.82	S	S	0.74	0.63	0.21
GSK2126458	0.03	0.17	R	R	0.75	0.45	0.44
Phenformin	0.07	0.10	R	R	0.76	0.43	0.39
MLN4924	0.07	0.14	R	R	0.69	0.35	0.31
Nilotinib	0.11	0.22	R	R	0.75	0.47	0.34
AP-24534	0.02	0.04	R	R	0.76	0.44	0.31
Embelin	0.01	0.07	R	R	0.73	0.31	0.35
AZD-0530	0.16	0.07	R	R	0.74	0.48	0.25
HG-5-113-01	0.01	0.08	R	R	0.76	0.37	0.23
VX-11e	0.68	0.08	S	R	0.74	0.42	0.31
Lenalidomide	0.07	0.22	R	R	0.75	0.47	0.40
MS-275	0.13	0.12	R	R	0.71	0.33	0.24
TGX221	0.09	0.05	R	R	0.71	0.43	0.39
OSI-906	0.18	0.66	R	S	0.74	0.43	0.21
BI-2536	0.10	0.11	R	R	0.76	0.40	0.25
XAV-939	0.05	0.04	R	R	0.70	0.40	0.33
GW-2580	0.29	0.46	R	S	0.72	0.38	0.36
PD-0332991	0.03	0.08	R	R	0.72	0.39	0.35
AZD7762	0.02	0.16	R	R	0.72	0.37	0.34
Tamoxifen	0.09	0.37	R	R	0.73	0.39	0.42
ABT-869	0.03	0.15	R	R	0.72	0.42	0.46
Mitomycin-C	0.02	0.17	R	R	0.72	0.36	0.34
SB52334	0.10	0.15	R	R	0.76	0.43	0.24
A-770041	0.38	0.26	S	R	0.71	0.68	0.28
Masitinib	0.11	0.05	R	R	0.74	0.41	0.42
KIN001-055	0.06	0.19	R	R	0.73	0.34	0.27
Temsirolimus	0.04	0.05	R	R	0.71	0.39	0.31
Axitinib	0.02	0.11	R	R	0.74	0.36	0.28
NU-7441	0.01	0.12	R	R	0.70	0.39	0.35
SB-715992	0.01	0.03	R	R	0.70	0.23	0.27
AG-014699	0.03	0.04	R	R	0.70	0.37	0.45
QL-VIII-58	0.02	0.05	R	R	0.65	0.29	0.32
Cetuximab	0.06	0.08	R	R	0.72	0.42	0.36
piperlongumine	0.07	0.07	R	R	0.70	0.30	0.14
JW-7-52-1	0.06	0.09	R	R	0.67	0.33	0.19
CMK	0.06	0.07	R	R	0.66	0.34	0.25
Gemcitabine	0.05	0.03	R	R	0.70	0.27	0.21
KU-55933	0.01	0.31	R	R	0.74	0.40	0.48
TW-37	0.08	0.03	R	R	0.69	0.33	0.23
Erlotinib	0.10	0.13	R	R	0.71	0.49	0.64
ABT-888	0.21	0.17	R	R	0.69	0.34	0.36
Cyclopamine	0.09	0.21	R	R	0.69	0.45	0.37

CGP-082996	0.04	0.13	R	R	0.68	0.31	0.17
SB-505124	0.01	0.25	R	R	0.67	0.33	0.26
SGC0946	0.18	0.11	R	R	0.67	0.35	0.34
BAY-61-3606	0.03	0.04	R	R	0.72	0.36	0.36
Sorafenib	0.11	0.03	R	R	0.69	0.34	0.34
HG-5-88-01	0.03	0.41	R	S	0.69	0.40	0.31
PD-173074	0.03	0.53	R	S	0.70	0.40	0.26
EHT-1864	0.04	0.18	R	R	0.69	0.29	0.28
rTRAIL	0.88	0.23	S	R	0.72	0.55	0.25
CX-5461	0.89	0.68	S	S	0.70	0.75	0.11
MG-132	0.06	0.12	R	R	0.64	0.32	0.25
Pyrimethamine	0.14	0.18	R	S	0.66	0.30	0.15
NSC-207895	0.46	0.04	S	R	0.72	0.29	0.12
BMS-754807	0.23	0.61	R	S	0.67	0.39	0.35
JNJ-26854165	0.08	0.05	R	R	0.70	0.29	0.21
CGP-60474	0.05	0.06	R	R	0.67	0.34	0.36
Pazopanib	0.05	0.09	R	R	0.64	0.36	0.38
Obatoclax-Mesylate	0.10	0.16	R	R	0.70	0.35	0.31
Thapsigargin	0.06	0.03	R	R	0.68	0.30	0.24
BMS-509744	0.06	0.06	R	R	0.70	0.34	0.27
YK-4-279	0.06	0.24	R	S	0.67	0.29	0.19
Z-LLNle-CHO	0.09	0.17	R	R	0.58	0.27	0.19
PFI-1	0.06	0.07	R	R	0.68	0.33	0.25
17-AAG	0.05	0.10	R	R	0.73	0.33	0.31
CP724714	0.12	0.37	R	S	0.64	0.31	0.21
Imatinib	0.06	0.18	R	R	0.67	0.37	0.31
LY317615	0.11	0.07	R	R	0.65	0.35	0.34
Sunitinib	0.05	0.04	R	R	0.76	0.48	0.46
LAQ824	0.28	0.06	R	R	0.65	0.34	0.43
Midostaurin	0.04	0.02	R	R	0.67	0.31	0.21
Bortezomib	0.14	0.12	R	R	0.69	0.34	0.36
VX-680	0.18	0.36	R	S	0.68	0.47	0.28
NVP-TAE684	0.11	0.04	R	R	0.65	0.32	0.31
Epothilone-B	0.02	0.13	R	R	0.59	0.20	0.20
S-Trityl-L-cysteine	0.05	0.04	R	R	0.76	0.40	0.30
FH535	0.11	0.09	R	R	0.65	0.33	0.26
PHA-665752	0.13	0.09	R	R	0.58	0.36	0.23
Paclitaxel	0.07	0.08	R	R	0.66	0.27	0.22
CCT018159	0.04	0.04	R	R	0.64	0.27	0.25
OSU-03012	0.04	0.38	R	S	0.67	0.30	0.19
Elesclomol	0.06	0.05	R	R	0.65	0.27	0.21
Salubrinal	0.07	0.15	R	R	0.67	0.33	0.37
RO-3306	0.16	0.09	R	R	0.63	0.33	0.32
Parthenolide	0.04	0.10	R	R	0.61	0.30	0.27
ZG-10	0.03	0.08	R	R	0.64	0.32	0.25
Roscovitine	0.09	0.04	R	R	0.66	0.32	0.22
GSK-650394	0.04	0.21	R	S	0.64	0.26	0.18
QL-XII-47	0.08	0.09	R	R	0.63	0.21	0.15
681640	0.10	0.18	R	R	0.64	0.26	0.27
AMG-706	0.11	0.05	R	R	0.58	0.28	0.16
XMD11-85h	0.07	0.17	R	R	0.65	0.44	0.50
GNF-2	0.25	0.19	S	R	0.56	0.30	0.24
Rapamycin	0.11	0.26	R	R	0.53	0.36	0.28
XMD8-85	0.08	0.14	R	R	0.68	0.29	0.21
Bexarotene	0.03	0.06	R	R	0.64	0.27	0.27
IOX2	0.03	0.17	R	R	0.59	0.31	0.35
SB-216763	0.29	0.08	S	R	0.65	0.37	0.28
FTI-277	0.06	0.11	R	R	0.61	0.33	0.25
JQ12	0.04	0.17	R	R	0.59	0.25	0.24
AUY922	0.01	0.07	R	R	0.60	0.20	0.18
Doxorubicin	0.02	0.07	R	R	0.62	0.29	0.18
MP470	0.10	0.50	R	S	0.64	0.33	0.15
Shikonin	0.05	0.08	R	R	0.59	0.26	0.28
PF-4708671	0.08	0.09	R	R	0.61	0.26	0.14
AKT-inhibitor-VIII	0.06	0.06	R	R	0.60	0.26	0.26
JNK-Inhibitor-VIII	0.32	0.06	S	R	0.65	0.32	0.25
KIN001-135	0.04	0.39	R	S	0.67	0.42	0.34
A-443654	0.05	0.06	R	R	0.61	0.29	0.22
Bryostatin-1	0.06	0.18	R	S	0.57	0.28	0.16
BIRB-0796	0.06	0.15	R	S	0.58	0.28	0.13
YM155	0.08	0.02	R	R	0.56	0.25	0.19
GW-441756	0.21	0.28	S	S	0.55	0.30	0.15
NSC-87877	0.06	0.12	R	S	0.57	0.28	0.10
FK866	0.01	0.04	R	S	0.54	0.04	0.04
CCT007093	0.16	0.34	R	S	0.58	0.30	0.27
SL-0101-1	0.12	0.35	R	R	0.62	0.32	0.37

GW843682X	0.07	0.11	R	R	0.61	0.21	0.12
LFM-A13	0.03	0.22	R	R	0.61	0.31	0.36
PF-562271	0.07	0.06	R	R	0.57	0.23	0.20
QS11	0.03	0.19	R	R	0.61	0.21	0.20
NVP-BEZ235	0.03	0.18	R	S	0.57	0.23	0.18
XMD8-92	0.04	0.17	R	R	0.54	0.23	0.23
KIN001-266	0.15	0.21	R	R	0.58	0.26	0.32
Tipifarnib	0.05	0.26	R	S	0.51	0.24	0.20
JNK-9L	0.08	0.06	R	R	0.51	0.20	0.29
Vinorelbine	0.01	0.20	R	S	0.58	0.21	0.14
AS601245	0.06	0.11	R	R	0.56	0.24	0.13
GSK-1904529A	0.17	0.26	S	S	0.46	0.23	0.02
**Avg.**					**0.72**	**0.42**	
